# Acid-sensing ion channel 1a regulates the specificity of reconsolidation of conditioned threat responses

**DOI:** 10.1172/jci.insight.155341

**Published:** 2022-02-22

**Authors:** Erin E. Koffman, Charles M. Kruse, Kritika Singh, Farzaneh Sadat Naghavi, Melissa A. Curtis, Jennifer Egbo, Mark Houdi, Boren Lin, Hui Lu, Jacek Debiec, Jianyang Du

**Affiliations:** 1 Department of Anatomy and Neurobiology, University of Tennessee Health Science Center, Memphis, Tennessee, USA.; 2 Department of Biological Sciences, University of Toledo, Toledo, Ohio, USA.; 3 Department of Pharmacology and Physiology, George Washington University School of Medicine, Washington DC, USA.; 4 Molecular & Behavioral Neuroscience Institute and Department of Psychiatry, University of Michigan, Ann Arbor, Michigan, USA.; 5 Neuroscience Institute, University of Tennessee Health Science Center, Memphis, Tennessee, USA.

**Keywords:** Neuroscience, Ion channels, Memory, Psychiatric diseases

## Abstract

Recent research on altering threat memory has focused on a reconsolidation window. During reconsolidation, threat memories are retrieved and become labile. Reconsolidation of distinct threat memories is synapse dependent, whereas the underlying regulatory mechanism of the specificity of reconsolidation is poorly understood. We designed a unique behavioral paradigm in which a distinct threat memory can be retrieved through the associated conditioned stimulus. In addition, we proposed a regulatory mechanism by which the activation of acid-sensing ion channels (ASICs) strengthens the distinct memory trace associated with the memory reconsolidation to determine its specificity. The activation of ASICs by CO_2_ inhalation, when paired with memory retrieval, triggers the reactivation of the distinct memory trace, resulting in greater memory lability. ASICs potentiate the memory trace by altering the amygdala-dependent synaptic transmission and plasticity at selectively targeted synapses. Our results suggest that inhaling CO_2_ during the retrieval event increases the lability of a threat memory through a synapse-specific reconsolidation process.

## Introduction

Recently, threat memory research in both rodents and humans has focused on a reconsolidation window after threat memory retrieval, in which the memory is labile and subject to intervention (1–4). Several studies have demonstrated that interrupting the updating process in reconsolidation aroused by retrieval prevents memory restorage, generating selective amnesia (5). Studies using rodent models have indicated that pharmacological intervention within the reconsolidation window successfully erases the retrieved specific threat memory (5–7). Recently, drug-free paradigms of intervention within reconsolidation have been proposed to prevent the return of threat memories in both rodents and humans (8–13).

Despite the importance of reconsolidation, much of its cellular mechanism is undiscovered. One of the known characteristics of reconsolidation is the specificity in which memory is reactivated specifically (14, 15). Retrieving auditory threat memories within the reconsolidation window requires the same conditioned stimulus (CS) to be presented in both retrieval and conditioning. Previous electrophysiology studies also demonstrated that retrieval triggers reconsolidation of memory and new learning by potentiating distinct synapses in the amygdala (3). More importantly, specifically enhancing a memory trace associated with threat conditioning increases the efficiency of memory retrieval, resulting in more efficient memory erasure after an extinction procedure is carried out. These prior discoveries led us to ask the following questions: Do the initial threat conditioning and retrieval events induce the same synapse, or do they induce separate synapses that share similar characteristics? Furthermore, is there a mechanism that allows us to manipulate reconsolidation more efficiently?

To answer these questions, we activated acid-sensing ion channels (ASICs) by CO_2_ inhalation, and then studied the effects on the erasure of threat memory. Through these experiments, we found ASICs and CO_2_ inhalation potentiate memory retrieval and increase memory lability (16). However, whether ASICs regulate the specificity of reconsolidation through activating the specific memory trace is still unclear and this question is outstanding. Furthermore, electrorheological and imaging studies in brain slices support the conclusion that the effects of ASICs on memory retrieval are particularly associated with a given memory. Our study proposes that CO_2_ inhalation paired with retrieval in the reconsolidation window triggers the original threat memory specifically, providing a unique angle to further study the mechanism underlying threat memory. Previous studies have suggested that protons are potential neurotransmitters (17–19) and their receptors, ASICs, serve as postsynaptic proton receptors that play key roles in neurotransmission and synaptic plasticity in the amygdala (20–22). Among the ASIC family members (ASIC1a, ASIC1b, ASIC2a, ASIC2b, ASIC3, and ASIC4), ASIC1a is predominantly and widely expressed in many areas of the brain, where it is associated with numerous brain functions and disorders (23–26). Disruption of ASIC1a affects synaptic transmission and plasticity (27–29), which suggests protons may sufficiently activate postsynaptic ASIC1a. In mice, disruption of ASIC1a activity alters neuronal activity and reduces threat response associated with threatening memories (30).

To study the effects of ASICs on the specificity of reconsolidation, we modified a threat conditioning paradigm that allows selective memory reactivation within the reconsolidation window from 2 distinct auditory threat memories (14). In this paradigm, 2 distinct CSs were paired with an unconditioned stimulus during conditioning. We hypothesized that if reconsolidation is specific, retrieving a memory with 1 CS followed by extinction can prevent the return of the memory. We also proposed that a separate CS (absent during retrieval) followed by extinction would not prevent the return of the memory. We tested this hypothesis using multiple behavioral, pharmacological, electrophysiological, and molecular methods, and our evidence supports that ASICs affect the reconsolidation window with specificity.

## Results

### CO_2_ selectively enhances the lability of auditory threat memory in the amygdala.

**Figure 1:**
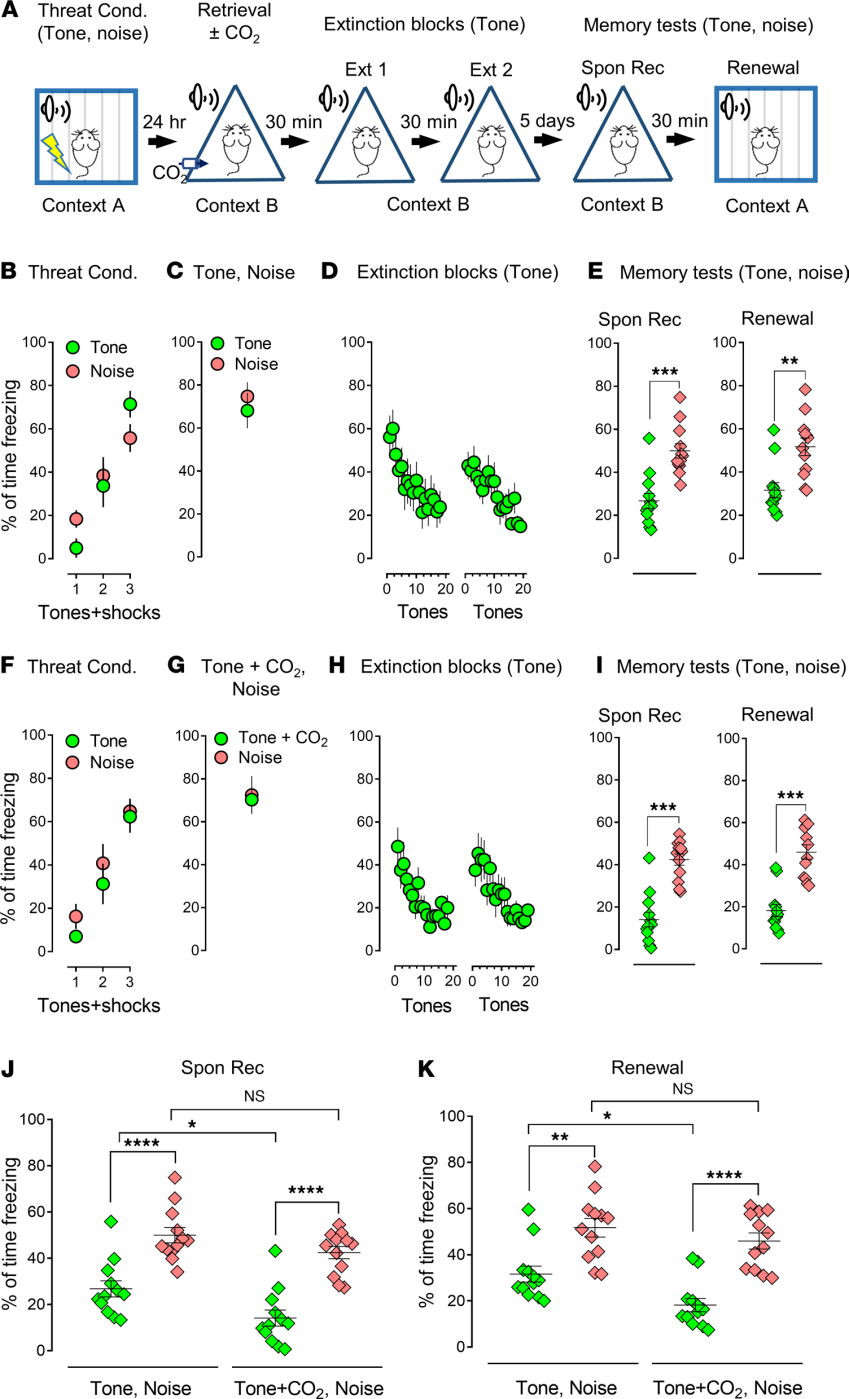
CO_2_ inhalation during a selective memory retrieval potentiates the effect of the extinction. (**A**) Schematic protocol for threat conditioning (Threat Cond.), memory retrieval, extinction (Ext), memory test–spontaneous recovery (Spon Rec), and renewal. On day 1, the mice were subjected to 3 pure tones and 3 white noises paired with 6 foot-shocks in context A. One day after, the mice were placed in context B and were subjected to both tone and noise as retrieval events. Thirty minutes after retrieval, the mice were treated with 2 blocks of extinctions with a pure tone as the CS. On day 7, Spon Rec and renewal were tested individually in context B and then context A. During each memory testing, 4 pure tones and 4 white noises were presented as CSs. (**B–E**) Data are presented by the percentage of freezing time during the CSs (tone and noise) in threat conditioning (**B**), retrievals (tone and noise) (**C**), 2 sections of extinction with tone (**D**), memory test of Spon Rec and renewal with tone and noise (**E**). (**F–I**) Data are presented by the percentage of freezing time during the CSs (tone and noise) in threat conditioning (**F**), retrievals (tone plus CO_2_ inhalation and noise) (**G**), 2 sections of extinction with tone (**H**), Spon Rec, and renewal with tone and noise (**I**). (**J** and **K**) Comparison data based on Spon Rec and renewal, respectively, from **E** and **I**. Data are mean ± SEM. *n* = 12 mice in each group. *P < 0.05, **P < 0.01, ***P < 0.001, ****P < 0.0001, by 2-tailed paired Student’s *t* test (**E** and **I**) or 1-way ANOVA with Tukey’s post hoc multiple-comparison test (**J** and **K**).

Previous studies have described a threat conditioning paradigm in which memory can be selectively reactivated and reconsolidated, suggesting synapse-specific reconsolidation of distinct threat memories in the amygdala (14, 15). We followed this paradigm, albeit with modifications (Figure 1A and Supplemental Figure 1A; supplemental material available online with this article; https://doi.org/10.1172/jci.insight.155341DS1). On day 1, we trained the animals with 2 distinct conditioned stimuli: 3 pure tones and 3 white noises paired with 1 foot-shock per stimuli as threat unconditioned stimulus (see the detailed description in Methods). We evaluated the outputs of the threat conditioning through the percentage of the freezing time within the time of CSs. The freezing was significantly increased after each of the 3 conditioned stimuli, indicating the mice were trained sufficiently under the designed condition (Figure 1B and Supplemental Figure 1B).

On day 2, the animals were placed into a new context (context B) and presented with 1 pure tone followed by a single noise (or vice versa) to retrieve the memory (Figure 1, A and C, and Supplemental Figure 1, A and C). The animals were then returned to their home cages; 30 minutes later, all mice underwent 2 blocks of extinctions in context B. Each extinction contained 20 pure tones (Figure 1D) or 20 white noises (Supplemental Figure 1D). At the end of the extinction procedure, the freezing dropped down to a low level (Figure 1D and Supplemental Figure 1D), suggesting that the extinction procedure was sufficient to suppress the memory. Five days later, the mice underwent spontaneous recovery (Spon Rec) (context B) and renewal (context A), respectively. Four tones and 4 noises were presented throughout the memory test (Figure 1A and Supplemental Figure 1A). The group that underwent extinction with a specific CS showed specificity in which freezing response was lowered after extinction (Figure 1E and Supplemental Figure 1E). For example, when the pure tone was presented during extinction, the percentage of freezing time in the memory test in the pure tone group (Spon Rec: 26.73% ± 3.5%; Renewal: 31.63% ± 3.4%) was lower than freezing in the noise group (Spon Rec: 49.98% ± 3.3%; Renewal: 51.73% ± 4.1%) (Figure 1E, Spon Rec: *P* *=* 0.0002, *t* = 5.573, degrees of freedom (df) = 11; Renewal: *P* *=* 0.0034, *t* = 3.721, df = 11, 2-tailed paired Student’s *t* test), and vice versa (Supplemental Figure 1E, Spon Rec: *P* *=* 0.0009, *t* = 4.533, df = 11; Renewal: *P* = 0.0005, *t* = 4.851, df = 11, 2-tailed paired Student’s *t* test). When retrieval was paired with 10% CO_2_ inhalation, memory erasure effects were enhanced (Figure 1, F–I, Spon Rec: *P* *=* 0.0001, *t* = 5.878, df = 11; Renewal: *P* = 0.0001, *t* = 5.802, df = 11, 2-tailed paired Student’s *t* test; Supplemental Figure 1, F–I, Spon Rec: *P* *=* 0.0009, *t* = 4.495, df = 11; Renewal: *P* *=* 0.0002, *t* = 5.569, df = 11, 2-tailed paired Student’s *t* test), and there was statistical significance between the groups with or without CO_2_ in both spontaneous recovery and renewal (Figure 1, J and K, green columns, Spon Rec: *P* *=* 0.0418, F [degrees of freedom in the numerator (DFn), degrees of freedom in the denominator (DFd)] = 0.04819 [3, 44]; Renewal: *P* *=* 0.0463, F [DFn, DFd] = 0.9269 [3, 44]; and Supplemental Figure 1, J and K, red columns, Spon Rec: *P* *=* 0.0478, F [DFn, DFd] = 4.556 [3, 44]; Renewal: *P* *=* 0.0435, F [DFn, DFd] = 1.419 [3, 44], 1-way ANOVA and Tukey’s post hoc multiple-comparison test). To further evaluate the specificity of the effects of reconsolidation on memory modifications, we designed another retrieval protocol in which we presented pure tone and white noise, either of them paired with 10% CO_2_ inhalation, followed by an unrelated extinction of CS, white noise, or pure tone, respectively (Supplemental Figure 2). Consistent with our expectations, CO_2_ did not boost the effects on the retrieved memory in the absence of paired extinction compared with the data in Figure 1 and Supplemental Figure 1 (Supplemental Figure 2, A–E, Spon Rec: *P* *=* 0.0049, *t* = 3.511, df = 11; Renewal: *P* *=* 0.0097, *t* = 3.125, df = 11; and Supplemental Figure 2, F–I, Spon Rec: *P =* 0.0126, *t* = 2.975, df = 11; Renewal: *P* *=* 0.0028, *t* = 3.825, df = 11, 2-tailed paired Student’s *t* test). When compared with the data in Figure 1, F–I, and Supplemental Figure 1, F–I, we found the application of 10% CO_2_ to the retrieval event failed to enhance the outcome after extinction, indicating a specificity of the CO_2_ effects. In all, our data suggest that memory encoded in the amygdala can be distinct, and the effects of CO_2_ on memory are specific.

**Figure 2:**
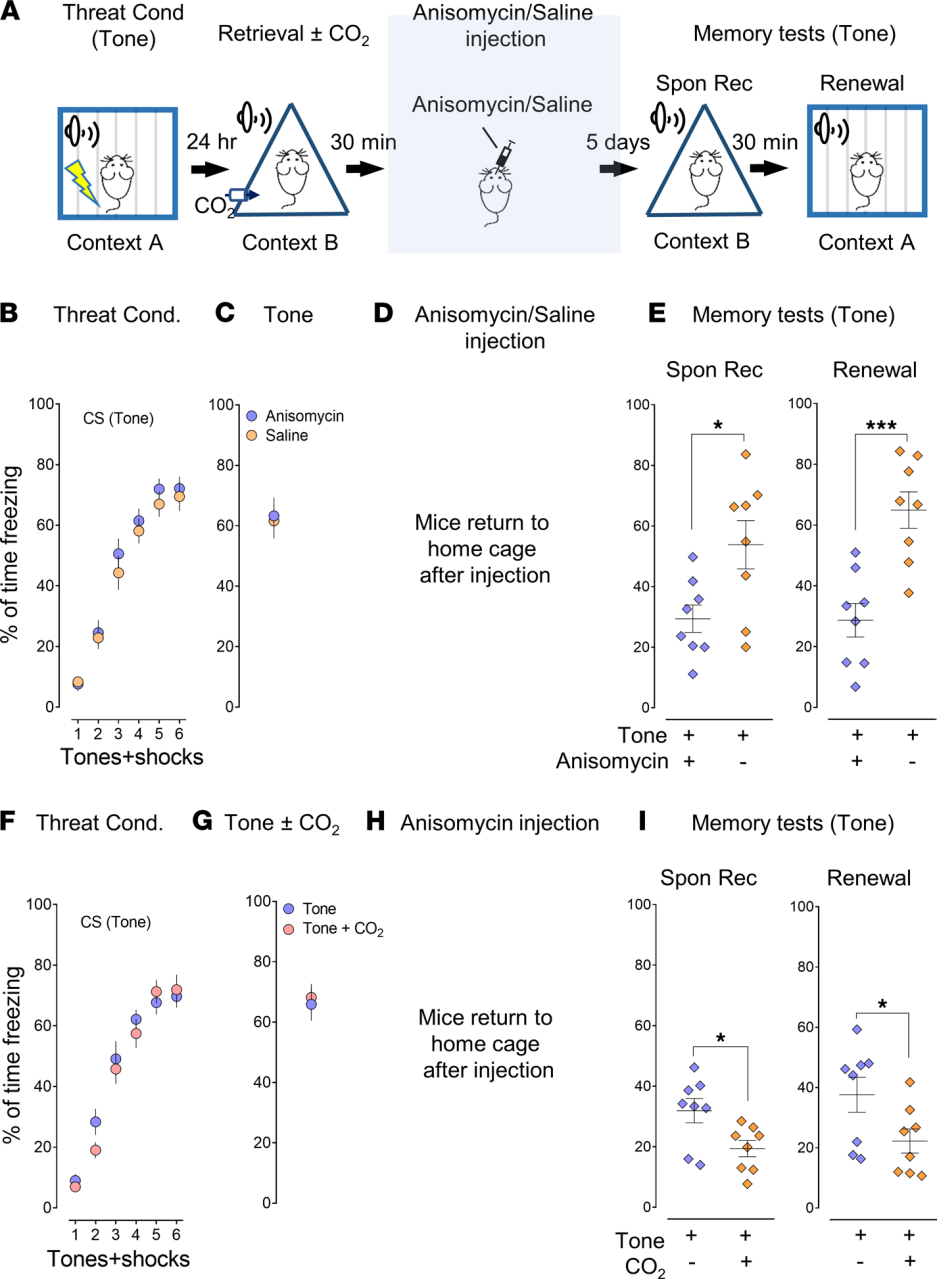
CO_2_ inhalation during memory retrieval potentiates the effect of the anisomycin. (**A**) Schematic protocol for threat conditioning, memory retrieval, anisomycin injection, memory test–Spon Rec, and renewal. Instead of the extinction procedure, 30 minutes after retrieval, the mice were infused with 62.5 μg/μL anisomycin or saline in each side of the amygdala and then returned to their home cages followed by Spon Rec and renewal test on day 7. (**B–E**) Data are presented by the percentage of freezing time during the tone presentation in threat conditioning (**B**), retrieval (tone) (**C**), saline or anisomycin infusion in the amygdala (**D**), Spon Rec and renewal test with tones (**E**). (**F–I**) Data are presented by the percentage of freezing time during the tone presentation in threat conditioning (**F**), retrieval (tone) with or without CO_2_ (**G**), anisomycin infusion (**H**), Spon Rec, and renewal test with tones (**I**). Data are mean ± SEM. *n* = 8 mice in each group. *P < 0.05, ***P < 0.001, by 2-tailed paired Student’s *t* test.

To focus on testing the specific effects of CO_2_ on retrieval, we replaced the extinction procedure with an injection of a eukaryotic protein synthesis inhibitor, anisomycin, to obliterate the threat memory (Figure 2A). Anisomycin, when injected bilaterally into the amygdala after retrieval, causes memory erasure compared with a saline injection (14). We conditioned the mice and retrieved the memory with pure tones (Figure 2, B and C) followed by anisomycin/saline injection (Figure 2D). Our experiments showed that anisomycin disrupted the memory during reconsolidation (Figure 2E, Spon Rec: *P* *=* 0.0453, *t* = 2.431, df = 7; Renewal: *P* *=* 0.0027, *t* = 4.527, df = 7, 2-tailed paired Student’s *t* test), and that memory retrieval was required for memory erasure with anisomycin injection (Supplemental Figure 3, A–E, Spon Rec: *P* *=* 0.0453, *t* = 2.431, df = 7; Renewal: *P* *=* 0.0027, *t* = 4.527, df = 7, 2-tailed paired Student’s *t* test). Consistent with the extinction results in Figure 1, when retrieval was paired with 10% CO_2_ inhalation, we found anisomycin reduced more threat response, further confirming that CO_2_ enhances memory lability specifically (Figure 2, F–I, Spon Rec: *P* *=* 0.0285, *t* = 2.751, df = 7; Renewal: *P* *=* 0.0223, *t* = 2.922, df = 7, 2-tailed paired Student’s *t* test). To exclude the possibility that anisomycin associates with one CS other than the other, as rigorous controls, we conditioned the mice with pure tone and white noise, carried out memory retrieval with both CSs, and found anisomycin had equal effects on memory in both tone and noise groups (Supplemental Figure 3, F–I, Spon Rec: *P* *=* 0.1480, *t* = 1.556, df = 11; Renewal: *P* *=* 0.0724, *t* = 1.987, df = 11; and Supplemental Figure 3, J–M, Spon Rec: *P* *=* 0.2178, *t* = 1.307, df = 11; Renewal: *P* *=* 0.3089, *t* = 1.067, df = 11, 2-tailed paired Student’s *t* test). When 10% CO_2_ was applied while the CSs were presented, the retrieval group paired with CO_2_ showed less freezing, regardless of the type of CS (pure tone or white noise) (Supplemental Figure 4, A–E, Spon Rec: *P* *=* 0.0462, *t* = 2.246, df = 11; Renewal: *P* *=* 0.0724, *t* = 1.987, df = 11; and Supplemental Figure 4, F–I, Spon Rec: *P* *=* 0.0341, *t* = 2.418, df = 11; Renewal: *P* *=* 0.0399, *t* = 2.329, df = 11, 2-tailed paired Student’s *t* test). As rigorous controls, we applied CO_2_ for both retrieval events together, and anisomycin decreased the freezing level in both groups, suggesting that CO_2_ had equal effects on both tone and noise (Supplemental Figure 4, J–M, Spon Rec: *P* *=* 0.0934, *t* = 1.837, df = 11; Renewal: *P* *=* 0.2210, *t* = 1.297, df=11; and Supplemental Figure 4, N–Q, Spon Rec: *P* *=* 0.1151, *t* = 1.711, df = 11; Renewal: *P* *=* 0.3142, *t* = 1.055, df = 11, 2-tailed paired Student’s *t* test). As another control, saline injection after retrieval and CO_2_ did not cause memory erasure, indicating that anisomycin was necessary to disrupt the reactivated memory (Supplemental Figure 5, A–E, Spon Rec: *P* *=* 0.3114, *t* = 1.061, df = 11; Renewal: *P* *=* 0.4839, *t* = 0.7245, df = 11; and Supplemental Figure 5, F–I, Spon Rec: *P* *=* 0.5385, *t* = 0.6349, df = 11; Renewal: *P* *=* 0.5262, *t* = 0.6546, df = 11, 2-tailed paired Student’s *t* test). Taken together, our data demonstrated that the effects of CO_2_ are specific to a distinct memory that is activated by a specific CS.

### The specific effects of CO_2_ on memory lability are ASIC dependent.

**Figure 3:**
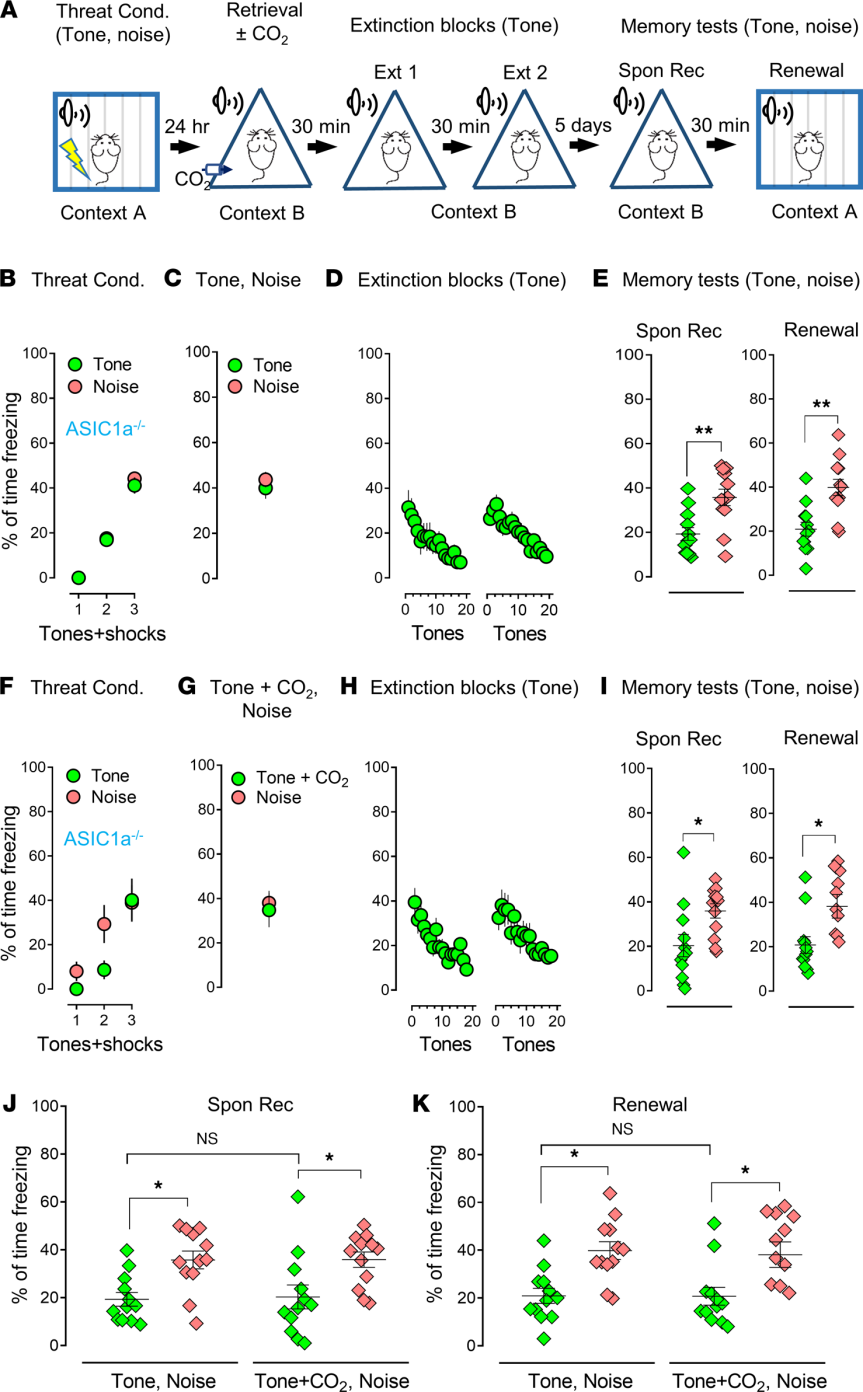
The effect of CO_2_ inhalation on selective memory retrieval is ASIC1a dependent. (**A**) Schematic of protocol for the threat conditioning, memory retrieval, extinction, memory test–Spon Rec, and renewal in ASIC1a^–/–^ mice. (**B**–**E**) Data in ASIC1a^–/–^ mice are presented by the percentage of freezing time during the CSs (tone and noise) in threat conditioning (**B**), retrievals (tone and noise) (**C**), 2 sections of extinction with tone (**D**), Spon Rec, and renewal with tone and noise (**E**). (**F**–**I**) Data in ASIC1a^–/–^ mice in threat conditioning (**F**), retrievals (pure tone plus 10% CO_2_ inhalation and white noise) (**G**), 2 sections of extinction with pure tone (**H**), Spon Rec, and renewal with tone and noise (**I**). (**J** and **K**) Comparison data based on Spon Rec and renewal, respectively, from **E** and **I**. Data are mean ± SEM. *n* = 12 mice in each group. *P < 0.05, **P < 0.01, ***P < 0.001, by 2-tailed paired Student’s *t* test (**E** and **I**) or 1-way ANOVA with Tukey’s post hoc multiple-comparison test (**J** and **K**).

**Figure 4:**
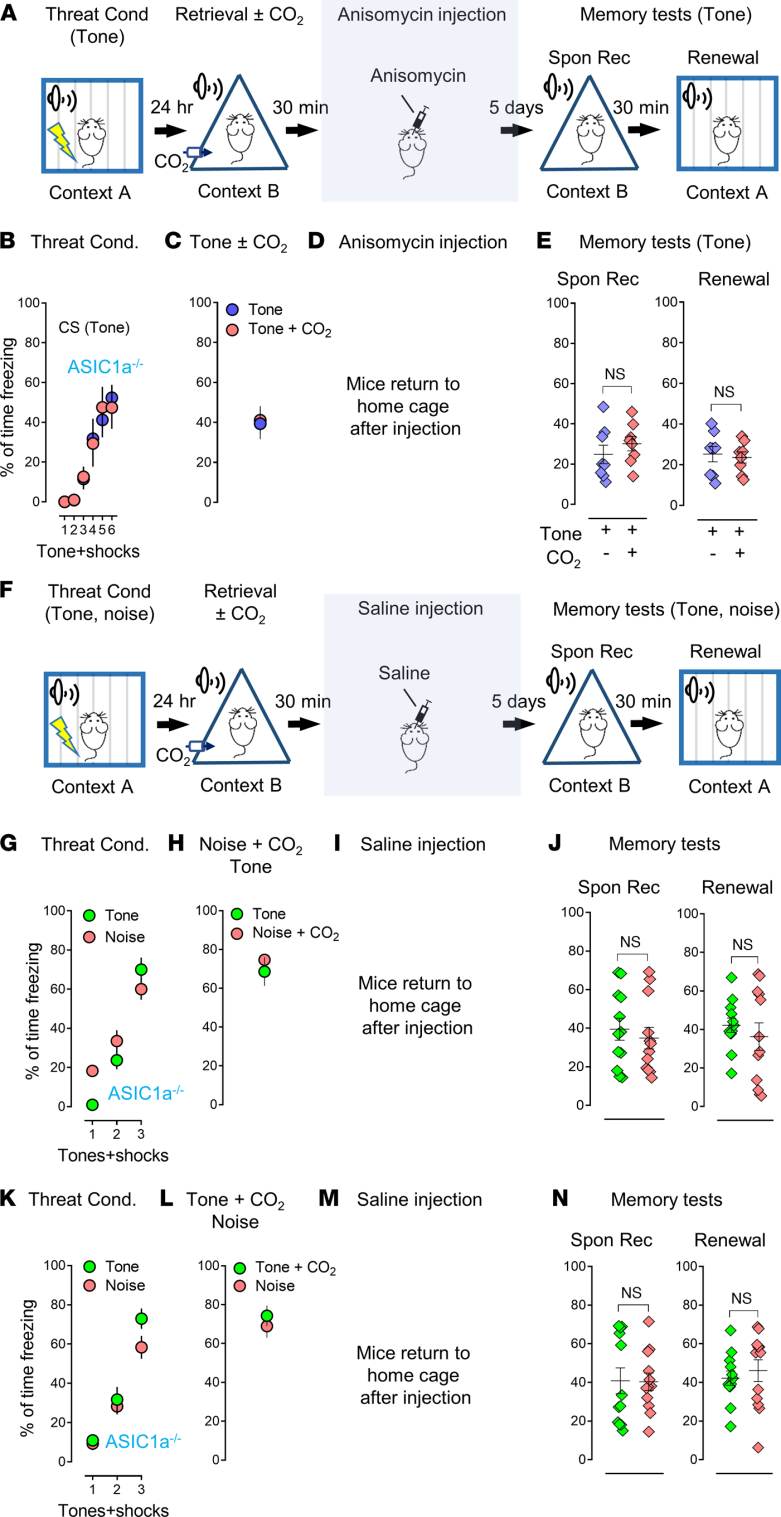
The CO_2_-potentiated anisomycin effects are ASIC1a dependent. (**A**) Schematic of protocol for the threat conditioning, memory retrieval (tone ± CO_2_), anisomycin injection, memory test–Spon Rec, and renewal. (**B–E**) Data in ASIC1a^–/–^ mice are presented by the percentage of freezing time during the tone presentation in threat conditioning (**B**), retrieval (tone) with or without CO_2_ (**C**), anisomycin infusion in the amygdala (**D**), Spon Rec, and renewal test with tones (**E**), *n* = 8 mice in each group. (**F**) Schematic protocol for threat conditioning, memory retrieval (tone and noise ± CO_2_), anisomycin injection, Spon Rec, and renewal. (**G**–**J**) Data are presented by the percentage of freezing time during the tone presentation in threat conditioning (**G**), retrieval (noise plus CO_2_ inhalation, then tone) (**H**), anisomycin infusion in the amygdala (**I**), Spon Rec and renewal test with tones (**J**), *n* = 12 mice in each group. (**K**–**N**) Data are presented by the percentage of freezing time during the tone presentation in threat conditioning (**K**), retrieval (tone) with or without CO_2_ (**L**), anisomycin infusion in the amygdala (**M**), Spon Rec and renewal test with tones (**N**), *n* = 12 mice in each group. Data are mean ± SEM. NS, not statistically significant by 2-tailed paired Student’s *t* test between groups.

We have previously found the effects of CO_2_ on memory retrieval to be ASIC dependent (16). However, it is still unknown if CO_2_ application to a specific memory trace affects an ASIC-dependent mechanism. To answer this question, we first performed distinct threat conditioning in ASIC1a^–/–^ mice with 3 pure tones and white noises on day 1 (Figure 3A), followed by a pure tone and white noise for retrieval on day 2. We performed extinctions with pure tones 30 minutes after retrieval. Five days later, we tested spontaneous recovery and renewal with 4 pure tones and white noises. Similar to the response we saw in ASIC1a^+/+^ mice, the freezing level in the pure tone group of ASIC1a^–/–^ mice was less than that in the white noise group (spontaneous recovery, 46% decrease; renewal, 47.5% decrease) (Figure 3, B–E, Spon Rec: *P* *=* 0.0016, *t* = 4.153, df = 11; Renewal: *P* *=* 0.0026, *t* = 3.862, df = 11, 2-tailed paired Student’s *t* test). When 10% CO_2_ inhalation was paired with pure tone in retrieval, we found that CO_2_ did not have additional effects on the memory with the specific CS in ASIC1a^–/–^ mice (spontaneous recovery, 43.4% decrease; renewal, 45.6% decrease) (Figure 3, F–I, Spon Rec: *P =* 0.0354, *t* = 2.397, df = 11; Renewal: *P* *=* 0.0215, *t* = 2.677, df = 11, 2-tailed paired Student’s *t* test), and there was no statistically significant difference between the groups with or without CO_2_ in both spontaneous recovery and renewal (Figure 3, J and K, green columns, Spon Rec: *P =* 0.9975, F [DFn, DFd] = 0.4967 [3, 44]; Renewal: *P* *>* 0.9999, F [DFn, DFd] = 1.367 [3, 44], 1-way ANOVA and Tukey’s post hoc multiple-comparison test). We had hypothesized that the effects of CO_2_ on memory retrieval would be ASIC dependent, and our data supported this prediction. We then replaced the extinction procedure with anisomycin to obliterate the threat memory (Figure 4A). The ASIC1a^–/–^ mice were conditioned with pure tones (Figure 4B) followed by a single tone as retrieval (Figure 4C). We then applied anisomycin infusions (Figure 4D) and tested the memory 5 days later (Figure 4E). Anisomycin dramatically reduced freezing in memory tests that followed, whereas pairing with CO_2_ in the CS did not cause an additional reduction in the ASIC1a^–/–^ mice, suggesting an ASIC dependency (Figure 4E, Spon Rec: *P* *=* 0.4687, *t* = 0.7661, df = 7; Renewal: *P* *=* 0.7400, *t* = 0.3453, df = 7, 2-tailed paired Student’s *t* test). We also presented 2 distinct CSs (pure tone and white noise) during conditioning and then retrieval, with or without 10% CO_2_, followed by anisomycin infusions in the ASIC1a^–/–^ mice (Figure 4F). Anisomycin dramatically reduced freezing in the memory test that followed, whereas pairing with CO_2_ in either CSs (pure tone or white noise) did not cause an additional reduction in the ASIC1a^–/–^ mice, suggesting an ASIC dependency (Figure 4, G–J, Spon Rec: *P* *=* 0.6245, *t* = 0.5036, df = 11; Renewal: *P* *=* 0.5081, *t* = 0.6840, df = 11; and Figure 4, K–N, Spon Rec: *P* *=* 0.9461, *t* = 0.06911, df = 11; Renewal: *P* *=* 0.5991, *t* = 0.5413, df = 11, 2-tailed paired Student’s *t* test).

**Figure 5:**
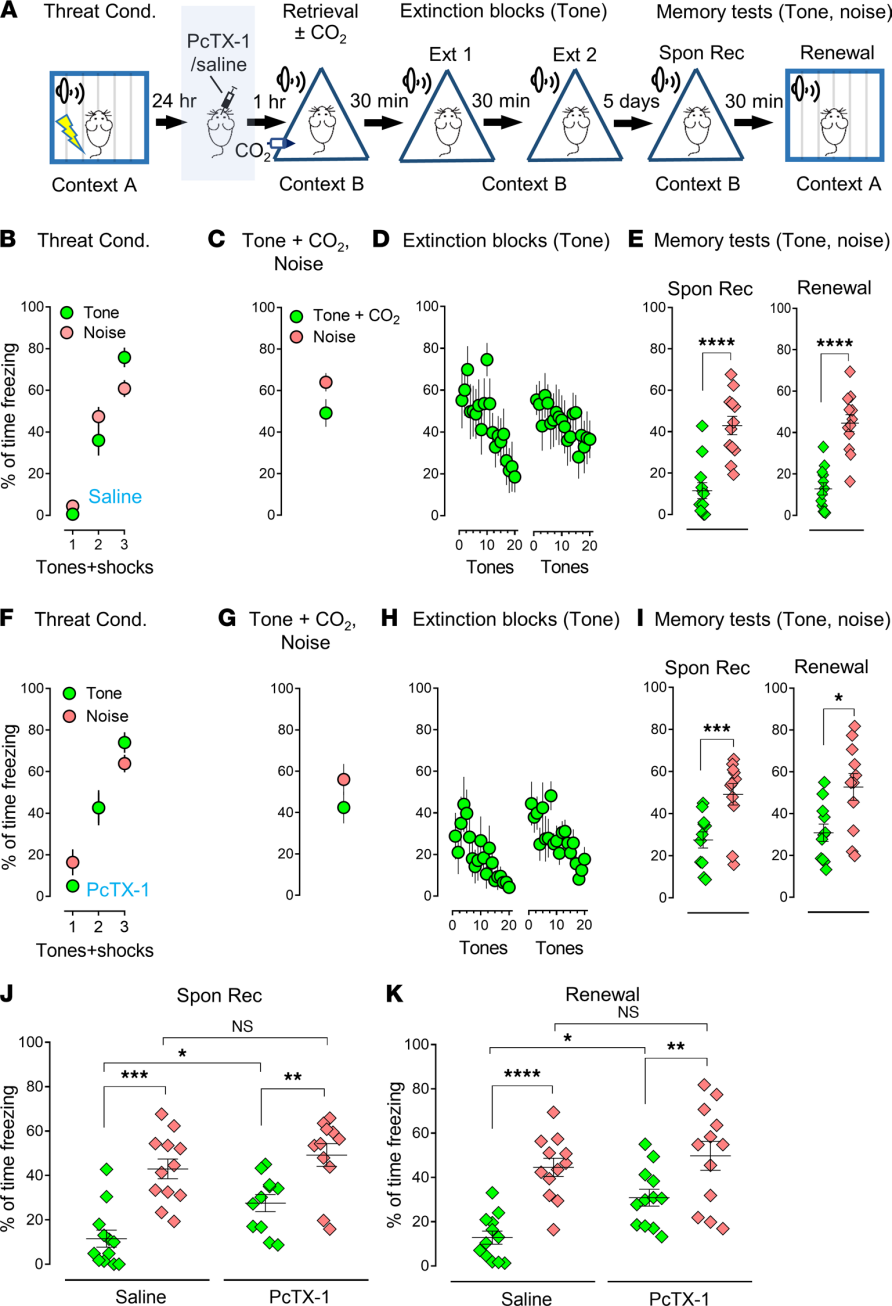
Blockage of ASIC1a in the amygdala reduces the CO_2_ effects on selective memory retrieval. (**A**) Schematic protocol for the threat conditioning, PcTX-1 injection, memory retrieval, extinction, memory test–Spon Rec, and renewal. One day after conditioning, the mice were injected with 100 nM PcTX-1 or saline; then the mice were placed in context B and subjected to both tone and noise as retrieval events with or without CO_2_ followed by extinction and memory test. (**B**–**E**) Data are presented by the percentage of freezing time during the CSs (tone and noise) in threat conditioning (**B**), retrievals (tone plus CO_2_ inhalation and noise) after saline injection in the amygdala (**C**), 2 sections of extinction with tone (**D**), Spon Rec and renewal with tone and noise (**E**), *n* = 12 mice in each group. (**F**–**I**) Data are presented by the percentage of freezing time during the CSs (tone and noise) in threat conditioning (**F**), retrievals (tone plus CO_2_ inhalation and noise) after PcTx-1 injection in the amygdala (**G**), 2 sections of extinction with tone (**H**), Spon Rec and renewal with tone and noise (**I**), *n* = 11 mice in each group. (**J** and **K**) Comparison data based on Spon Rec and renewal, respectively, from **E** and **I**. Data are mean ± SEM. *P < 0.05, **P < 0.01, ***P < 0.001, ****P < 0.0001, by 2-tailed paired Student’s *t* test (**E** and **I**) or 1-way ANOVA with Tukey’s post hoc multiple-comparison test (**J** and **K**).

To provide evidence that an acute ASIC1a blockage was able to eliminate the effects of CO_2_, we injected the selective ASIC1a inhibitor, 100 nM Psalmotoxin-1 (PcTX-1), into the lateral amygdala bilaterally 1 hour before the application of CO_2_ to the retrieval (Figure 5A). Our data suggest that compared with the saline injection group (Figure 5, B–E, Spon Rec: *P* *<* 0.0001, *t* = 6.383, df = 11; Renewal: *P* *<* 0.0001, *t* = 7.497, df = 11, 2-tailed paired Student’s *t* test), inhibiting ASIC1a by PcTX-1 (Figure 5, F–I, Spon Rec: *P* *=* 0.0008, *t* = 4.767, df = 10; Renewal: *P* *=* 0.0249, *t* = 2.637, df = 10, 2-tailed paired Student’s *t* test) significantly reduced the CO_2_ effects on memory retrieval; statistical analysis in the spontaneous recovery and renewal groups supported this conclusion (Figure 5, J and K, green columns, Spon Rec: *P* *=* 0.0360, F [DFn, DFd] = 0.3499 [3, 42]; Renewal: *P =* 0.0364, F [DFn, DFd] = 2.448 [3, 44], 1-way ANOVA and Tukey’s post hoc multiple-comparison test). This pattern of findings suggests that the effects of CO_2_ on specific memory traces are ASIC dependent.

### Activation of ASICs through CO_2_ inhalation alters reconsolidation of distinct memory through alteration of AMPARs.

**Figure 6:**
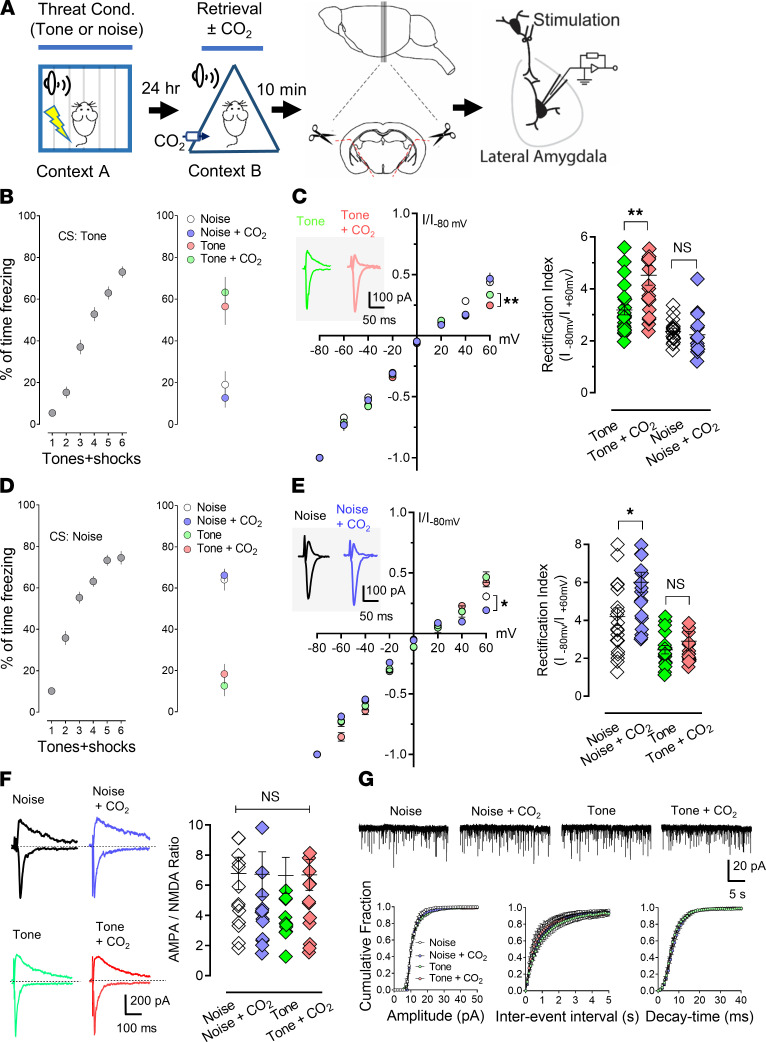
CO_2_ inhalation during a selective memory retrieval enhances the retrieval-dependent AMPAR current rectification. (**A**) Schematic protocol. On day 1, the animals underwent 6 CSs (tones or noses) paired with 6 foot-shocks in context A. On day 2, the mice were divided into 4 groups for the retrieval: pure tone only; pure tone plus 10% CO_2_ inhalation; white noise only; white noise + 10% CO_2_ inhalation. Ten minutes after retrieval, the brain slices were dissected, and AMPAR current was recorded in the pyramidal neurons in the lateral amygdala through stimulation of thalamic input. (**B**–**E**) Mice underwent 6 pure tones (**B**) or 6 noises (**D**) in threat conditioning. Data are presented by the percentage of freezing time during the tone presentation in threat conditioning, retrieval (noise plus CO_2_ inhalation, then tone). (**C** and **E**) Left, AMPAR current-voltage relationships in the recorded neurons. Insets show an example of the AMPAR/EPSCs in –80mV and +60mV. Right, AMPAR rectification index (I–80 mV/I+60 mV). Data are mean ± SEM. *n* = 20–26 for each group, *n* = 15–23 cells/4–5 mice for each group. (**F**) Left, examples of EPSC recordings of AMPAR/EPSCs (–80mV) and NMDAR/EPSCs (+60mV). Right, AMPAR/NMDAR EPSC ratios. Current amplitudes were measured 70 ms after onset. *n* = 16 cells/4 mice for each group. (**G**) mEPSC recordings from the neurons after retrieval. Upper, representative mEPSC traces from different groups. Lower, cumulative distributions of mEPSC amplitudes, inter-event intervals, and decay times, *n* = 12 cells/4 mice for each group. Data are mean ± SEM. *P < 0.05, **P < 0.01, by ANOVA with Tukey’s post hoc multiple-comparison test.

AMPARs are glutamatergic receptors that have crucial roles in modulating memory retrieval and destabilization (8, 11, 31–33). Previous studies suggest that the exchange of Ca^2+^-impermeable AMPARs (CI-AMPARs) for Ca^2+^-permeable AMPARs (CP-AMPARs) occurs after retrieval (11, 34). According to a previous study, 10% CO_2_ inhalation paired with retrieval induces a stronger current rectification of AMPARs (the signature of CP-AMPARs) than in the retrieval-alone group, indicating a greater exchange of AMPARs (16). Interestingly, no further enhancement was observed in the ASIC1a^–/–^ brain slices, indicating that the effect of CO_2_ inhalation on AMPAR exchange is ASIC dependent (16). To further study whether CO_2_ specifically alters the AMPAR exchange in retrieval, we designed an experiment to separate the threat conditioning from retrieval and measure the rectification of AMPARs (Figure 6A). To study this, we conditioned the mice with 6 pure tones on day 1 (Figure 6B). On day 2, the mice were divided into 4 groups based on retrieval conditions — the first group received pure tone only; the second group pure tone plus 10% CO_2_ inhalation; the third group white noise only; and the fourth group received white noise plus 10% CO_2_ inhalation (Figure 6B). Ten minutes after retrieval, we dissected brain slices, and AMPAR current was recorded in the pyramidal neurons in the lateral amygdala through stimulation of thalamic inputs (Figure 6A). Rectification, a signature of CP-AMPARs, was compared among all groups. Consistent with earlier reports, pure tone retrieval increased current rectification (11, 34), and CO_2_ paired with pure tone retrieval caused stronger rectification (Figure 6C, *P* *=* 0.0012, F [DFn, DFd] = 4.558 [3, 69], 1-way ANOVA and Tukey’s post hoc multiple-comparison test). However, when white noise was presented as the retrieval event, both white noise and white noise plus CO_2_ failed to cause a significant rectification compared with the pure tone group (Figure 6C, *P =* 0.9881, F [DFn, DFd] = 4.558 [3, 69], 1-way ANOVA and Tukey’s post hoc multiple-comparison test). This data supports our prediction that CO_2_ was specific to a reactivated memory trace. To control for possible effects stemming from order of presentation of CSs, we switched over the pure tone and white noise in the threat conditioning and retrieval. Similar results were observed that confirmed the effects of CO_2_ were not artificial (Figure 6, D and E, noise versus noise + CO_2_ groups, *P* *=* 0.0223, and tone versus tone + CO_2_ groups, *P* *=* 0.9089, F [DFn, DFd] = 2.669 [3, 69], 1-way ANOVA and Tukey’s post hoc multiple-comparison test).

We then asked whether synaptic strength changed with the application of an unrelated retrieval CS and CO_2_. Previous studies have found the ratio of AMPAR/EPSC (excitatory postsynaptic current) to NMDAR/EPSC might represent the strength of the synapse (35). Previous studies have also reported that the AMPAR/NMDAR/EPSC ratios increased after threat conditioning, whereas retrieval did not potentiate further increase, suggesting that memory retrieval did not alter the synaptic strength (11, 34). Our previous studies also indicated that CO_2_ inhalation during memory retrieval did not strengthen the synapse in the amygdala (16). We further tested whether the pairing of CO_2_ inhalation with the specific retrieval CS altered the strength of a synapse. Currents were recorded at –80mV for AMPAR/EPSCs and +60mV for NMDAR/EPSCs. Our data suggest that retrieval plus CO_2_ inhalation did not change the AMPAR/NMDAR/EPSC ratio (Figure 6F, *P* *>* 0.9999, among groups, F [DFn, DFd] = 0.1351 [3, 57], 1-way ANOVA and Tukey’s post hoc multiple-comparison test). Moreover, the characteristics of miniature EPSCs (mEPSCs), including amplitude, frequency, and decay times, were not altered (Figure 6G). In addition, the pairing of CO_2_ with another unrelated CS in retrieval did not change the strength of the synapse (Figure 6, F and G). These data suggest that the effects of CO_2_ inhalation on specific memory retrieval enhance the destabilization of the synapse associated with the original memory without changing the AMPAR/NMDAR ratio. These data are also consistent with previous studies showing memory retrieval triggers the destabilization of the synapse that encodes the original memory through converting CI-AMPARs to CP-AMPARs without changing AMPAR current amplitude (11, 34).

### The effects of CO_2_ inhalation on distinct memory trace.

**Figure 7:**
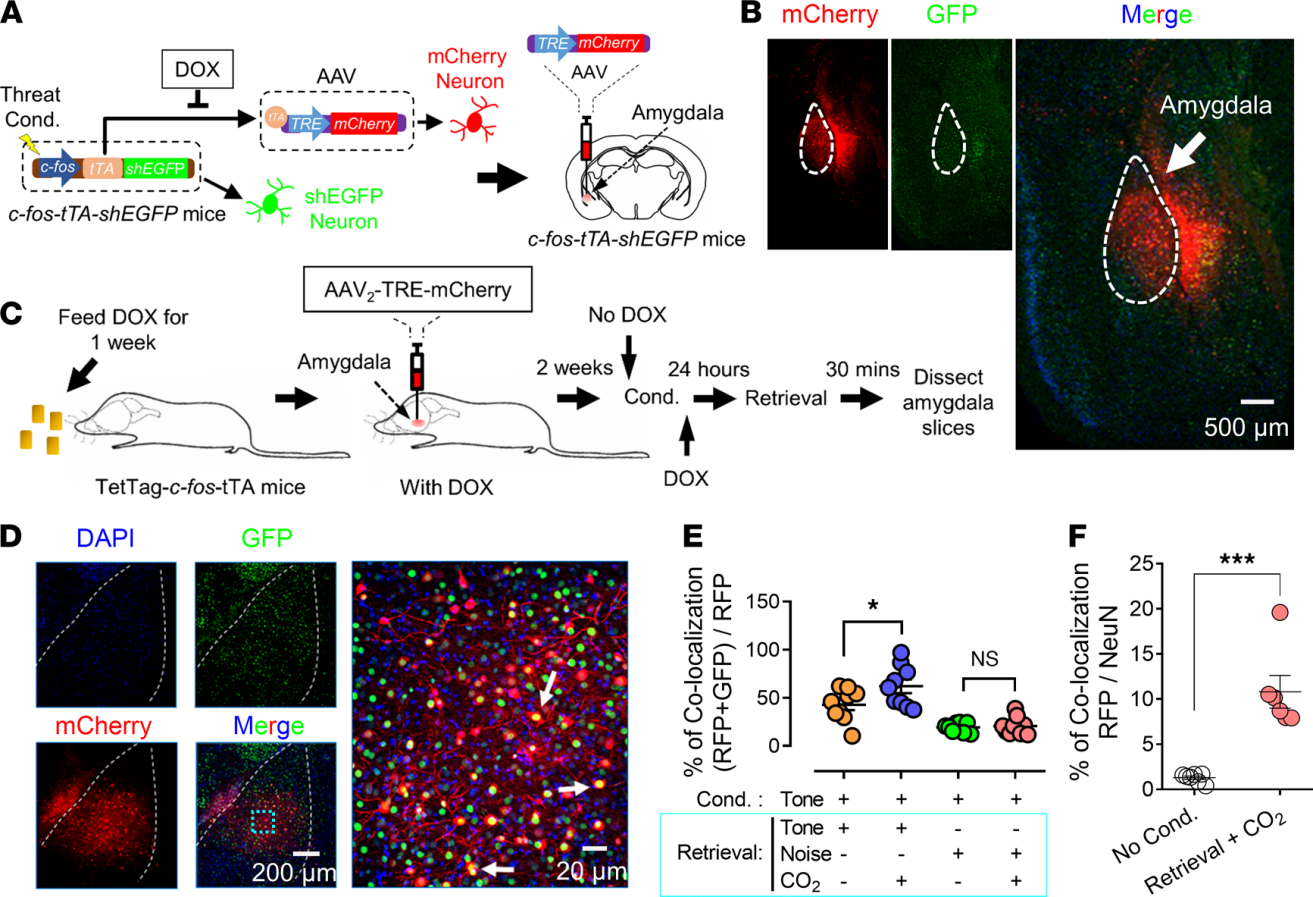
CO_2_ inhalation during a selective memory retrieval enhances the retrieval-related memory trace. (**A**) Schematic showing the c-Fos-tTA-GFP mouse system combined with an AAV_2_-mCherry to label a specific memory trace. (**B**) An example image showing the efficiency of the expression of GFP and mCherry in the amygdala (scale bar: 500 μm). (**C**) The procedure of threat conditioning, memory retrieval, and memory trace labeling using the system in **A**. (**D**) Left, representative images of the neurons labeled by mCherry, GFP, and DAPI (scale bar: 200 μm); right, the enlarged area from the “merge” image showing the overlapping expression of mCherry and GFP neurons. The overlapping neurons indicate their “consanguinity” in the same memory trace (scale bar: 20 μm). (**E**) Summarized data are the percentage of the overlapping expression of mCherry and GFP neurons in different behavior groups. All mice underwent threat conditioning with a tone as the CS. One day later, the mice were separated into 4 groups for retrieval experiment. Data are mean ± SEM. *n* = 9 slices/3 mice for each group. *P < 0.05, by 1-way ANOVA with Tukey’s post hoc multiple-comparison test. (**F**) Control experiment showing the expression of mCherry with or without DOX as well as with or without threat conditioning. Data are mean ± SEM. *n* = 7 slices/3 mice for each group. ***P < 0.001 by unpaired 2-tailed Student’s *t* test.

Our previous studies indicate that CO_2_ enhances memory trace associated with threat conditioning (16). In this experiment, we examined the mechanism behind the specificity of CO_2_ effects on memory traces. We used a c-Fos-tTA-GFP mouse system combined with an AAV_2_-mCherry to label a specific memory trace. The Fos promoter in transgenic mice was activated by behavioral activities, followed by the shGFP expression in the cells. When the AAV_2_-mCherry virus was injected into the brain, the activation of c-FOS also induced the expression of mCherry. When the mice were fed a doxycycline (DOX) diet, mCherry expression was interrupted. Using the TetTag-c-fos–driven-GFP mouse model, neurons in the amygdala involved in memory trace after threat conditioning can be labeled with a long-lasting mCherry fluorescent protein through virus injection (AAV_2_-TRE-mCherry) and the neuron in the retrieval trace can be labeled with a shEGFP (Figure 7, A and B) (see the details in Methods) (16, 36). The overlapped labeling (yellow) represents the neurons in the same memory trace of threat and retrieval (16, 37, 38). In this experiment, the mice were first conditioned with pure tone, activating the associated neurons that were labeled with mCherry (Figure 7C). Immediately after threat conditioning, the mice were fed a DOX diet, thereby preventing further mCherry labeling. On day 2, the mice were divided into 2 groups: 1 group of mice was presented with a single pure tone to retrieve the memory, another was presented with white noise. The shEGFP was labeled after the retrieval. Thirty minutes after the retrieval event, we sliced the amygdala and imaged shEGFP- and mCherry-positive cells (Figure 7D). Compared with the pure tone threat conditioning/pure tone retrieval group (same memory trace of threat conditioning and retrieval), inhalation of CO_2_ in the pure tone threat conditioning/white noise retrieval group did not result in an increase of neurons positive for expression of both mCherry-positive and shEGFP-positive neurons (overlapped labeling, yellow in Figure 7D, and Figure 7E, tone conditioning, tone versus tone + CO_2_ groups, *P* *=* 0.0450, and tone conditioning, noise versus tone + CO_2_ groups, *P* *=* 0.9971, F [DFn, DFd] = 5.138 [3, 31], 1-way ANOVA and Tukey’s post hoc multiple-comparison test). Control experiments to identify the efficiency of the threat conditioning on the expression of mCherry on the cells were performed (Figure 7F, *P* *=* 0.0001, *t* = 5.675, df = 11, 2-tailed unpaired Student’s *t* test). These findings indicated that CO_2_, when paired with retrieval, only enhanced the memory trace that had been reactivated; CO_2_ paired with unrelated retrieval cues did not affect the original memory trace. These findings suggest a specific effect of CO_2_ on the memory trace.

We then examined the effects of CO_2_ on dendritic spine morphology after memory retrieval. Spine morphology has been widely indicated in the mechanism of synaptic plasticity (39–41). Dendritic spines are the primary target of neurotransmission input in the CNS (42), and their density and structure provide the basis for physiological changes in synaptic efficacy that underlie learning and memory (43). Spine formation and plasticity are regulated by many conditions, including exterior stimulation and behavior (44). We hypothesized that CO_2_ inhalation during retrieval alters both structure and plasticity of dendritic spines. The molecular mechanism by which CO_2_ regulates spine plasticity may explain how CO_2_ converts memory into the labile stage.

**Figure 8:**
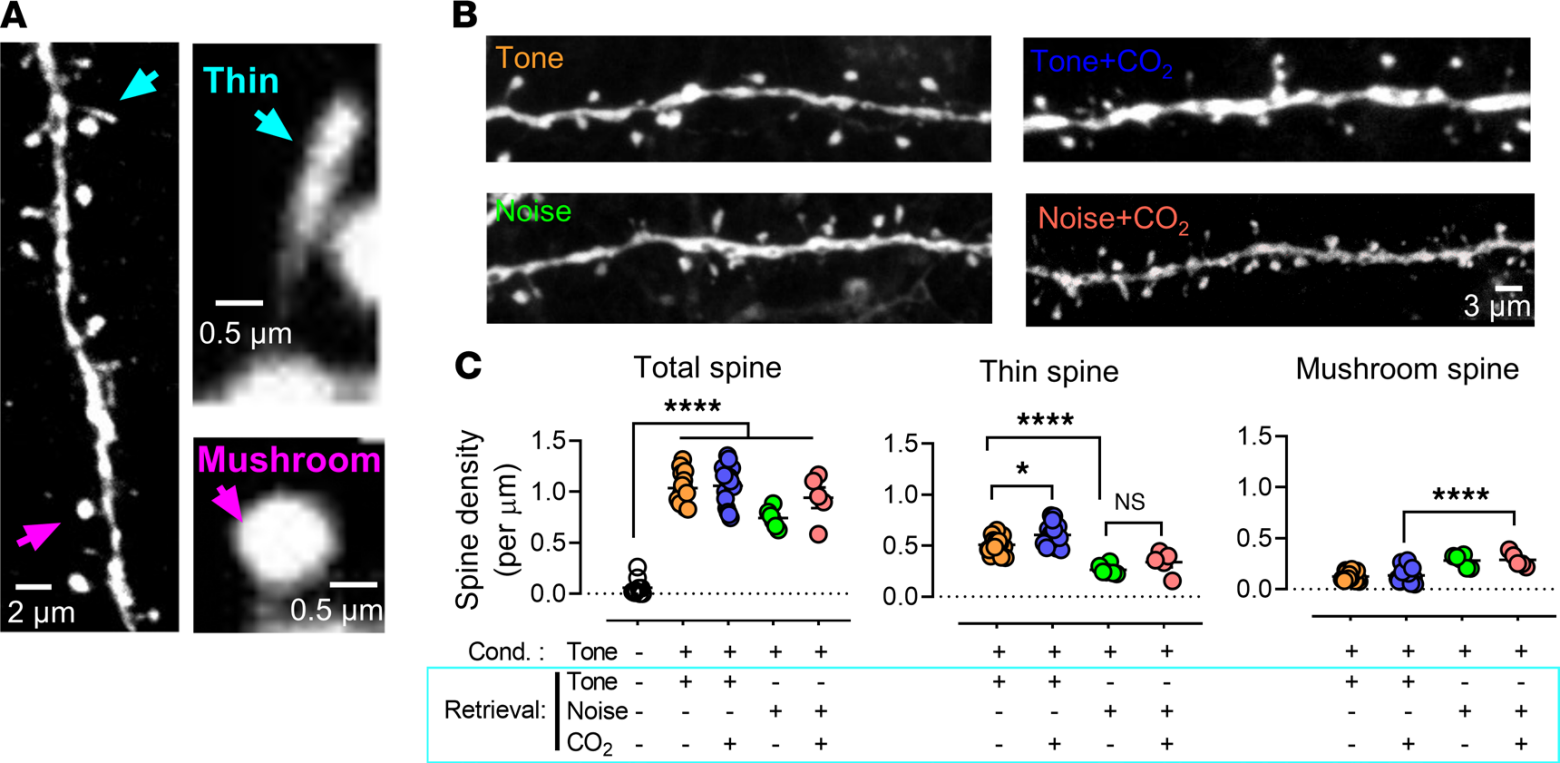
CO_2_ inhalation during a selective memory retrieval enhances the dendritic spine turnover. (**A**) Left, a representative image showing the spine morphology in the mCherry and GFP colocalized neurons (scale bar: 2 μm). The mature spines were categorized as “mushroom” spines and the immature spines were categorized as “thin” spines; right, an enlarged image showing the details of mushroom and thin spines (scale bar: 0.5 μm). (**B**) Representative images of the spine structures in different animal groups (scale bar: 20 μm). (**C**) Summarized data of the spine densities of mushroom spines, thin spines, and total spines in the different groups. Data are mean ± SEM. *n* = 10–16 slices/4 mice for each group. *P < 0.05, ***P < 0.001, ****P < 0.0001, by 1-way ANOVA with Tukey’s post hoc multiple-comparison test.

Using the TetTag-c-fos–driven-GFP mouse model in Figure 7, we imaged spine structure and assessed spine density and morphology in overlapping neurons of the amygdala in each group (pure tone threat conditioning, pure tone retrieval, pure tone threat conditioning, and white noise retrieval). Mature spines — most of which display “mushroom-like” morphology — have more stable postsynaptic structures enriched in AMPARs. In contrast, immature spines with a “thin-like” morphology are unstable postsynaptic structures that have the transitional ability. Immature dendritic spines are thought to be responsible for synaptic plasticity because they have the potential for strengthening (45). The categories of spines were identified based on the parameters in the previous studies (Figure 8, A and B) (see details in Methods) (39, 46). The behavior procedure is described in Figure 7C, in which the animals underwent threat conditioning with a tone as a CS followed by a retrieval on day 2 with tone or noise. We found increased spine numbers after threat conditioning, indicating increasing synaptic strength. There was no additional increase in the density of spines in all groups, suggesting that retrieval and CO_2_ inhalation did not change the synaptic strength (Figure 8C, *P* *<* 0.0001, F [DFn, DFd] = 0.8803 [4, 47], 1-way ANOVA and Tukey’s post hoc multiple-comparison test).

We further analyzed spine subtypes as described in the experimental procedures. We examined the ratio of the number of thin “immature” spines to the total number of spines to determine potential plasticity within the synapse (45). We examined the ratio of the number of thin spines to the total number of spines. When the retrieval group (tone) was paired with CO_2_, we found an additional increase of thin spines compared with the retrieval group alone (Figure 8C, *P* *=* 0.0276, F [DFn, DFd] = 1.350 [3, 38], 1-way ANOVA and Tukey’s post hoc multiple-comparison test). This finding suggests that CO_2_ paired with retrieval might boost synaptic plasticity compared with memory retrieval alone. Consistently, the mushroom spine numbers decreased in the tone and CO_2_ paired retrieval groups, suggesting a higher turnover rate after memory retrieval (Figure 8C). However, when the mice were threat-trained with the pure tone but presented with white noise in retrieval (a generated unrelated CS), we found that with or without CO_2_ inhalation, the thin spine number did not increase compared with those trained and retrieved with pure tone (Figure 8C, *P =* 0.5661, F [DFn, DFd] = 1.350 [3, 38], 1-way ANOVA and Tukey’s post hoc multiple-comparison test). This finding supports the specific effect of CO_2_ on the memory trace.

## Discussion

A newly acquired threat memory is labile and can be easily disrupted before it is transformed into a long-term stable state (47). An existing memory, when reactivated, may become labile again during a short postreactivation period known as the reconsolidation window (48). Previous studies using auditory threat conditioning found that a retrieval event utilizing a single tone CS renders the memory labile during the reconsolidation window (8). During this reconsolidation window, memory is sensitive to the updating processes that may either enhance or weaken the original memory (16, 49, 50). The reconsolidation window offers an opportunity to determine the mechanisms underlying the lability of an existing memory.

Threat memory reconsolidation is selective, and the reactivated memories are stable and resistant to disruption. Interrupting the retrieved memory in the reconsolidation window is a sufficient way to interrupt the long-term or short-term memory (8, 11). However, the lack of valuable tools to potentiate the lability of the retrieved memory in the reconsolidation window enhances the interruption of the memory. Our current work intends to address this issue. We found that the effects of ASICs and CO_2_ on memory reconsolidation and memory trace were specific to the reactivated conditioned cue. Besides manipulating the memory lability in the reconsolidation window, the memory extinction process is another selective and critical process to suppress memories. In the clinic, both reconsolidation and extinction have been proposed as treatment models of anxiety disorders.

We then asked about the cellular mechanisms by which ASIC1a and CO_2_ regulate the specificity of reconsolidation of the original memory. Previous research studying the mechanism of retrieval of threat memories has revealed the rapid and transient exchange from CI-AMPARs to CP-AMPARs in the lateral amygdala synapses (11, 34) after presenting the CS. However, we do not know whether the exchange of AMPARs is key for the specificity of reconsolidation. To address this issue, we conditioned the mice with a pure tone CS and a white noise followed by reactivating the memory exclusively with the pure tone with or without CO_2_ inhalation. Consistent with our earlier findings (16), we observed that CO_2_ increases AMPAR exchange when it is paired with retrieval. When mice were presented with an unrelated CS (white noise here) during the reconsolidation window, CO_2_ did not alter the AMPAR exchange, suggesting the effects of CO_2_ on memory trace are memory specific. In addition, synaptic strength (ratio of AMPAR/NMDAR and amplitude of the mEPSCs) was not altered while applying CO_2_ within the specific reconsolidation window, with or without combining with the memory trace. The exchange of CI-AMPARs to CP-AMPARs indicated a synaptic plasticity change when the memory trace was reactivated exclusively. Moreover, we found that the total number of spines did not change with or without CO_2_ inhalation when activating the exclusive memory trace, which suggests the strength of the synapse in a memory trace does not change, further supporting the reconsolidation specificity hypothesis. In addition, thin spine density significantly increased when the memory trace was reactivated exclusively with the CO_2_ inhalation, suggesting that CO_2_ changes plasticity when inducing the exclusive reconsolidation. On the other hand, when an unrelated CS was presented during the reconsolidation window, no additional increase of immature spine density occurred. This finding indicates no synaptic plasticity change in the nonspecific memory trace. Thus, we can conclude from our findings that the effects of CO_2_ on memory trace are specific.

We also questioned whether CO_2_ affects all types of memory reconsolidation. Memory reconsolidation has been discovered in diverse species, ranging from *C. elegans* to humans (51). Reconsolidation occurs in varied memories, including hippocampal-, amygdala-, and cortical-dependent memories. This has been seen in emotional, appetitive, neutral memories; simple and complex task memories; and drug-paired, spatial, and motor memories (52). Although we have provided evidence that ASICs and CO_2_ indeed act with specificity on a threat memory trace, we cannot predict the possibility that CO_2_ might trigger specific effects on other types of memories, and this is a promising area for future study. In addition, reconsolidation can be evoked by many types of CSs and unconditioned stimuli as retrieval triggers. The mechanism of CS- and unconditioned stimulus–induced memory reconsolidation can be diverse and might accompany differing network activation, neurotransmission, and temporal progression. Whether CO_2_ and ASIC1a have similar effects on different retrieval events is unknown, which is another promising area for study.

Also, we cannot exclude the possibility that CO_2_ might trigger specific effects on memory through other targets. For instance, CO_2_ inhalation increases cerebral blood flow and arterial blood pressure and may affect brain functions, such as cognition (53). Although no direct evidence supports the possibility that increased cerebral blood flow and arterial blood pressure affect learning and memory, future studies will have to test this probability underlying the specificity of CO_2_ on a memory.

In conclusion, the effects of CO_2_ on threat memory reconsolidation were found to be exclusively under the reactivation of the original memory. Our research tests the hypothesis that protons are neurotransmitters that activate the postsynaptic proton receptors, ASICs, to manipulate memory updates. This noninvasive, drug-free methodology is innovative, efficacious, and might be valuable for translation to clinical use. As a result, this research may lead to an effective complementary treatment for many mental health–related disorders for which efficient treatments are lacking. We hope this research will lead to new areas of inquiry into CO_2_-related mechanisms that underlie memory modification and lead to the development of novel therapies for anxiety disorders such as PTSD.

## Methods

### Mice

For our experiment, we used male and female mice between 10 and 14 weeks of age. Mice were derived from a congenic C57BL/6 background: WT, ASIC1a-KO (ASIC1a^–/–^), and TetTag-c-fos-tTA mice. The ASIC1a^–/–^ homozygous mouse line was refreshed every 5–6 generations by backcrossing to C57BL/6J mice (The Jackson Laboratory strain number 000664). Mice (ASIC1a^+/+^ and ASIC1a^–/–^) generated from these crosses were used in behavioral assays, including littermates and non-littermates (Supplemental Figure 6). TetTag-cFos-tTA mice were obtained from The Jackson Laboratory (strain number 018306) and crossed with C57BL/6J mice. Mice carrying the Fos-tTA transgene were selected; Fos-tTA mice have a Fos promoter driving expression of nuclear-localized, 2-hour half-life EGFP (shEGFP) (16, 36, 38). The Fos promoter also drives the expression of tetracycline transactivator (tTA), which binds to the tetracycline-responsive element (TRE) site on an injected recombinant adeno-associated virus, AAV_2_-TRE-mCherry virus, resulting in the expression of mCherry (16, 36). The binding of the tTA to the TRE site is inhibited by DOX. Inhibition of tTA binding prevents target gene expression (37, 38, 54). Experimental mice were maintained on a standard 12-hour light/12-hour dark cycle and received standard chow and water ad libitum. Animal care and procedures met the NIH standards.

### Threat conditioning, retrieval, extinction, and memory test

#### Standard CS auditory threat conditioning, retrieval, extinction, and memory test.

On day 1 in a curated environment (context A), the experimental mice were presented with 6 pure tones (80 dB, 2 kHz, 20 seconds each) paired with 6 foot-shocks, 1 shock at the end of each tone (0.7 mA, 2 seconds). The interval between each tone was 100 seconds. On day 2, the mice were placed into a new environment (context B) and habituated for 4 minutes. Mice then inhaled either unaltered air or air containing 10% CO_2_ for 7 minutes. Five minutes after inhalation of CO_2_ or air began, mice were presented with one 20-second pure tone to retrieve the memory. The mice were returned to their home cages; 30 minutes later, the mice returned to the retrieval chamber (context B) and underwent 2 rounds of extinctions. In the first round of extinction, mice were exposed to 20 pure tones with an interval between tones of 100 seconds. Mice were returned to their home cages; 30 minutes later, the mice went through the extinction protocol again with 20 pure tones. On day 7, the mice were tested to see if their threat response would recur via spontaneous recovery in context B with 4 pure tones. Thirty minutes after spontaneous recovery, the mice were returned to the original context of the threat memory, context A, in a recovery protocol with 4 pure tones. Freezing behavior in mice (the absence of movement beyond respiration) is used as a measure of threat response. To evaluate the outcomes of freezing behavior in mice, the percentage of time during CS presentation spent in freezing was scored automatically using VideoFreeze software (Med Associates Inc.). In the spontaneous recovery and renewal tests, outcomes of the percentage of time freezing were averaged from each of the 4 CSs.

#### Two distinct CS auditory threat conditioning, retrieval, extinction, and memory test.

This procedure was used to test the specificity of the effects of CO_2_ on memory retrieval. The context settings and parameters are similar to previously described standard 1 CS auditory threat conditioning (16). In contrast to the above experiment, the mice were presented with 3 pure tones (80 dB, 2 kHz, 20 seconds each) that alternated with 3 white noises (60 dB, 2 kHz, 20 seconds each); all 6 stimuli were paired with foot-shocks. On day 2, the mice inhaled either unaltered air or air containing 10% CO_2_ for 7 minutes. Five minutes after inhalation of CO_2_ or air began, the mice underwent retrieval with 1 single pure tone followed by 1 white noise with or without CO_2_. This was followed 30 minutes later by 2 sections of extinctions with either pure tones or white noises. On day 7, the mice were tested via spontaneous recovery and renewal protocols with 4 pure tones and 4 white noises, respectively.

#### Two distinct CS auditory threat conditioning, retrieval, anisomycin, and memory tests.

In a series of experiments, the standard extinction procedure was replaced with amygdala infusion of a eukaryotic protein synthesis blocker, anisomycin (detailed in the surgery procedure below). In brief, the cannula was implanted on the amygdala 4–7 days before the behavioral experiments. On day 1, the mice were subjected to the threat conditioning described in the above experiment. On day 2, 30 minutes after retrieval, the mice were infused with 62.5 μg/μL anisomycin via the cannula in the lateral nuclei of the amygdala (LA) bilaterally and returned to their home cages (14). On day 7, the mice were tested via spontaneous recovery and renewal as described in the above experiment 2.

### Surgery and chemical infusion

For the cannula placement procedure, mice were anesthetized with isoflurane through an anesthetic vaporizer, secured to the stereotaxic instrument, and the cannula made from a 25-gauge needle was inserted bilaterally into LA and basolateral amygdala (relative to bregma: –1.2 mm anteroposterior; ±3.5 mm mediolateral; –4.3 mm dorsoventral) (16, 36). Dental cement secured the cannula and bone anchor screw in place. Mice recovered for 4–5 days before any subsequent testing was carried out. A 10 μL Hamilton syringe connected to a 30-gauge injector was inserted 1 mm past the cannula tip to inject the selective ASIC1a inhibitor, PcTX-1 (100 nM, Allomone Labs) or anisomycin (62.5 μg/μL, Cayman Chemical). The chemicals were diluted in 1 μL artificial cerebrospinal fluid (ACSF), pH 7.3, and injected over 5 minutes each side. The injection sites were mapped postmortem by sectioning the brain (10 μm coronal) and examining Cresyl violet staining using a Nissl Stain Kit (FD Neuro Technologies). Only animals that had a correctly placed cannula in the amygdala were included in the statistical analysis.

### Brain slice preparation and patch-clamp recording of amygdala neurons

Ten minutes after the memory retrieval experiment ended, mice were euthanized with overdosed isoflurane, and whole brains were dissected into preoxygenated (5% CO_2_ and 95% O_2_) ice-cold high-sucrose dissection solution containing (in mM): 205 sucrose, 5 KCl, 1.25 NaH_2_PO_4_, 5 MgSO_4_, 26 NaHCO_3_, 1 CaCl_2_, and 25 glucose (16). A vibratome sliced brains coronally into 300 μm sections that were maintained in normal ACSF containing (in mM): 115 NaCl, 2.5 KCl, 2 CaCl_2_, 1 MgCl_2_, 1.25 NaH_2_PO_4_, 11 glucose, 25 NaHCO_3_ bubbled with 95% O_2_/5% CO_2_, pH 7.35 at 20°C–22°C. Slices were incubated in the ACSF at least 1 hour before recording. For experiments, individual slices were transferred to a submersion-recording chamber and were continuously perfused with the 5% CO_2_/95% O_2_ solution (~3.0 mL/min) at room temperature (20°C–22°C).

As we described previously (16), pyramidal neurons in the lateral amygdala were studied using whole-cell patch-clamp recordings. The pipette solution contained (in mM): 135 KSO_3_CH_3_, 5 NaCl, 10 HEPES, 4 MgATP, 0.3 Na_3_GTP, 0.5 K-EGTA (mOsm = 290, adjusted to pH 7.25 with KOH). The pipette resistance (measured in the bath solution) was 3–5 MΩ. High-resistance (>1 GΩ) seals were formed in voltage-clamp mode. Picrotoxin (100 μM) was added to the ACSF throughout the recordings to yield excitatory responses. In AMPAR current rectification experiments, we applied D-APV (100 μM) to block NMDAR/EPSCs. The peak amplitude of ESPCs was measured to determine current rectification. The amplitude was measured ranging from –80 mV to +60 mV in 20 mV steps. The peak amplitude of EPSCs at –80 mV and +60 mV was measured for the rectification index. In EPSC ratio experiments, neurons were measured at –80 mV to record AMPAR/EPSCs and were measured at +60 mV to record NMDAR/EPSCs. To determine the AMPAR/NMDAR ratio, we measured the peak amplitude of EPSCs at –80 mV and the peak amplitude of EPSCs at +60 mV at 70 ms after onset. Data were acquired at 10 kHz using Multiclamp 700B and pClamp 10.1. The mEPSC events (>5 pA) were analyzed in Clampfit 10.1. The decay time (τ) of mEPSCs was fitted to an exponential using Clampfit 10.1.

### Immunohistochemistry and cell counting

The AAV-TRE-mCherry plasmid was obtained from the laboratory of Susumu Tonegawa at MIT (Cambridge, Massachusetts, USA) and Steve Ramirez at Boston University (Boston, Massachusetts, USA) (37, 38) and was used to produce AAV_2_ by the University of Iowa Gene Transfer Vector Core. For 1 week leading up to virus microinjection, TetTag Fos-tTA mice were fed with food containing 40 mg/kg DOX. We used a 10 μL Hamilton microsyringe and a WPI microsyringe pump to inject the virus (0.5 μL of 1.45E +1 2 viral genomes/mL of AAV_2_-TRE-mCherry) bilaterally into the amygdala (relative to bregma: –1.2 mm anteroposterior; ±3.5 mm mediolateral; –4.3 mm dorsoventral), as described previously (16, 36). For a 2-week window between surgery and behavior training, mice were housed and fed with a DOX-containing diet. The DOX-containing diet was ceased 24 hours before threat conditioning began on day 1 (replaced by a regular diet), and then immediately restarted afterward. Thirty minutes after retrieval on day 2, the mice were euthanized according to protocol. We used transcardial perfusion with 4% paraformaldehyde (PFA) to fix whole brains, followed by continued fixation in 4% PFA at 4°C for 24 hours (39). After perfusion, we used a vibratome (Leica VT-1000S) to dissect 50 μm amygdala coronal slices, which were collected in ice-cold PBS. To complete immunostaining, slices were placed in Superblock solution (Thermo Fisher Scientific) plus 0.2% Triton X-100 for 1 hour and incubated with primary antibodies (1:1000 dilution) at 4°C for 24 hours (16). We used the following primary antibodies: rabbit polyclonal IgG anti-RFP (Rockland, 600-401-379); chicken IgY anti-GFP (Thermo Fisher Scientific, A10262), and mouse anti-NeuN (MilliporeSigma, MAB377X) (37, 38). We then washed and incubated slices for 1 hour with secondary antibodies: Alexa Fluor 488 goat anti-chicken IgG (H+L) (Molecular Probes, A-11039), Alexa Fluor 568 goat anti-rabbit IgG (H+L) (Molecular Probes, A-21429), and Alexa Fluor 647 goat anti-mouse IgG (H+L) (Thermo Fisher Scientific, A-21235, 1:200 dilution). Vectashield H-1500 (Vector Laboratories, H-1500) was used to mount slices; confocal microscopy was used to view the slices. We used ImageJ (NIH) to analyze dendritic spine morphology. Thin, mushroom, and stubby spines were categorized based on the following parameters: a) mushroom spines: head-to-neck diameter ratio greater than 1.1:1 and spine head diameter greater than 0.35 μm; b); thin spines: head-to-neck diameter ratio greater than 1.1:1 and spine head diameter greater than 0.35 μm or spine head-to-neck diameter ratios less than 1.1:1 and spine length-to-neck diameter greater than 2.5 μm; c); stubby spines: spine head-to-neck diameter ratios less than 1.1:1 and spine length-to-neck diameter of 2.5 μm or less (39, 46).

### Statistics

A 2-tailed paired Student’s *t* test or an unpaired Student’s *t* test was used to compare results between 2 groups. One-way ANOVA and Tukey’s post hoc multiple-comparison test were used for the statistical comparison of more than 2 groups. *P* less than 0.05 was considered statistically significant, and we did not exclude potential outliers from our data. GraphPad Prism 8 was used to analyze statistical data, which is presented as mean ± SEM. Sample sizes (*n*) are indicated in the figure legends, and data are reported as biological replicates (data from different mice, different brain slices). Each group contained tissues pooled from 4–5 mice. Because of variable behavior within groups, we used sample sizes of 10–16 mice per experimental group as we previously described in earlier experiments (16). In behavioral studies, we typically studied groups with 4 randomly assigned animals per group, as our recording equipment allowed us to record 4 separate animal cages simultaneously. The experiments were repeated with another set of 4 animals until we reached the target number of experimental mice per group. Experimentation groups were repeated in this manner so that each animal had the same controlled environment — the same time of day and with similar handling, habituation, and processes.

### Study approval

The University of Tennessee Health Science Center Laboratory Animal Care Unit (protocol 19-0112) and University of Toledo IACUC (protocol 108791) approved all procedures.

## Author contributions

J Du, J Debiec, EEK, CMK, and HL conceived the project. J Du, EEK, CMK, J Debiec, and HL designed the experiments. EEK, CMK, JE, MH, and BL performed the behavior experiments. KS, MAC, and FSN performed the spine morphology experiments and data analysis. FSN and J Du performed the patch-clamp experiments and data analysis. EEK and J Du wrote the manuscript. All authors reviewed and edited the manuscript.

## Supplementary Material

Supplemental data

## Version 1 01/13/2022

In-Press Preview

## Version 2 02/22/2022

Electronic publication
