# Supplementary material for: Acid-sensing ion channel 1a regulates the specificity of reconsolidation of conditioned threat responses
Source: JCI Insight. 2022 Feb 22;7(4):e155341. doi: 10.1172/jci.insight.155341 (PMC8876458; doi:10.1172/jci.insight.155341)
Supplement: Supplemental data [file jciinsight-7-155341-s135.pdf]

Supplemental Figure 1

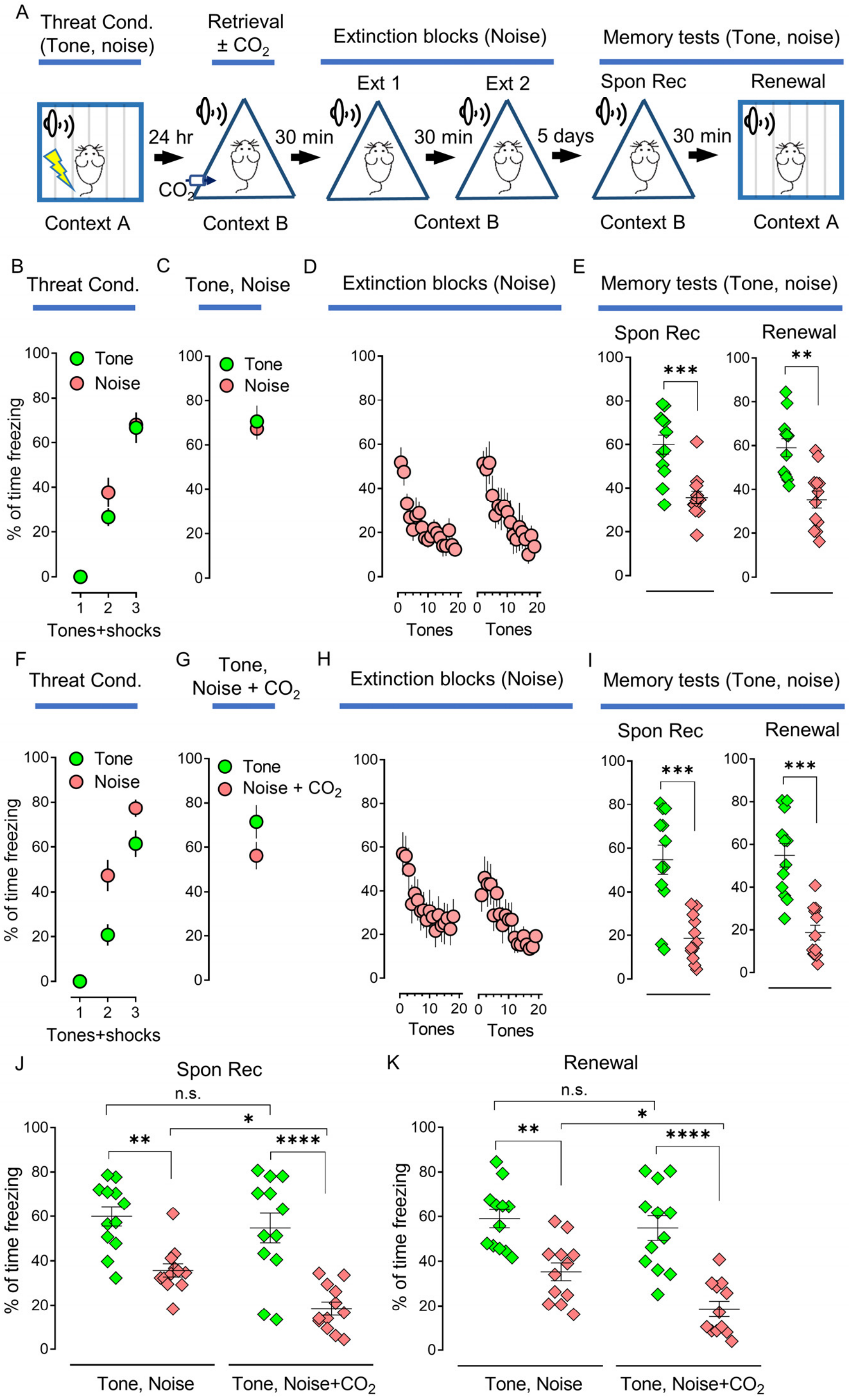

Supplemental Figure 1 White noise was an appropriate successful CS to involve use in studying the effects of CO2 effects on threat conditioning and retrieval. (A) Schematic protocol for the threat conditioning (pure tone and white noise), memory retrieval (pure tone and white noise), extinction (white noise), and memory test-Spon Rec and renewal. **(B-E)** Data are presented by the percentage of freezing time during the CSs (tone and noise) in threat conditioning **(B)**, retrievals (tone and noise) **(C)**, two sections of extinction with white noise **(D)**, Spon Rec, and

renewal with tone and noise **(E)**. **(F-I)** Data are presented by the percentage of freezing time during the CSs (tone and noise) in threat conditioning **(F)**, retrievals (pure tone and white noise plus CO_2 inhalation) **(G)**, two sections of extinction with white noise **(H)**, Spon Rec and renewal with tone and noise **(I)**. **(J-K)**, comparison data based on Spon Rec and renewal respectively from panels **E** and **I**. Data are mean ± SEM. n = 12 mice in each group. ‘n.s.’ indicates not statistically significant. * indicates $p < 0.05$, ** indicates $p < 0.01$, *** indicates $p < 0.001$, *** indicates $p < 0.0001$, by two-tailed paired Student’s t-test (panel E, I) or one-way ANOVA with Tukey’s posthoc multiple comparisons (panel J, K).

Supplemental Figure 2

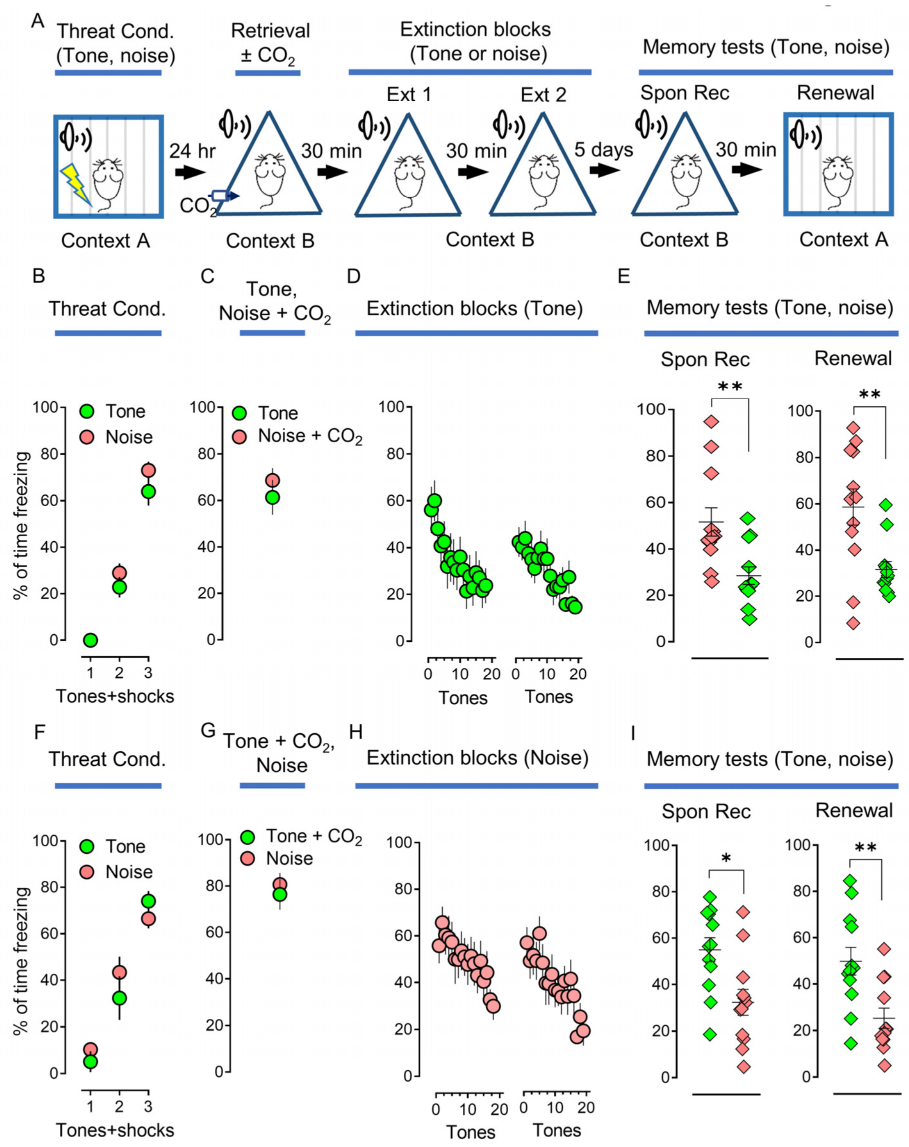

Supplemental Figure 2 CO_2 inhalation does not boost extinction when pairs with an unrelated CS during memory retrieval. (A) Schematic protocol for the threat conditioning (pure tone and white noise), memory retrieval (pure tone and white noise), extinction (white noise), and memory test-Spon Rec and renewal. **(B-E)** Data are presented by the percentage of freezing time during the CSs (tone and noise) in threat conditioning **(B)**, retrievals (pure tone and white noise plus CO_2 inhalation) **(C)**, two sections of extinction with pure tone **(D)**, Spon Rec and renewal with tone and noise, Blue arrow and % indicated the difference (decreases) between the tone and noise groups **(E)**. **(F-I)** Data are presented by the percentage of freezing time during the CSs (tone and noise) in threat conditioning **(F)**, retrievals (pure tone plus CO_2 inhalation and white noise) **(G)**, two sections of extinction with white noise **(H)**, Spon Rec and renewal with tone and noise, Blue arrow and % indicated the difference (decreases) between the tone and noise groups. **(I)**. Data are mean ± SEM. n = 12 mice in each group. * indicates $p < 0.05$ by two-tailed paired Student's t-test.

Supplemental Figure 3

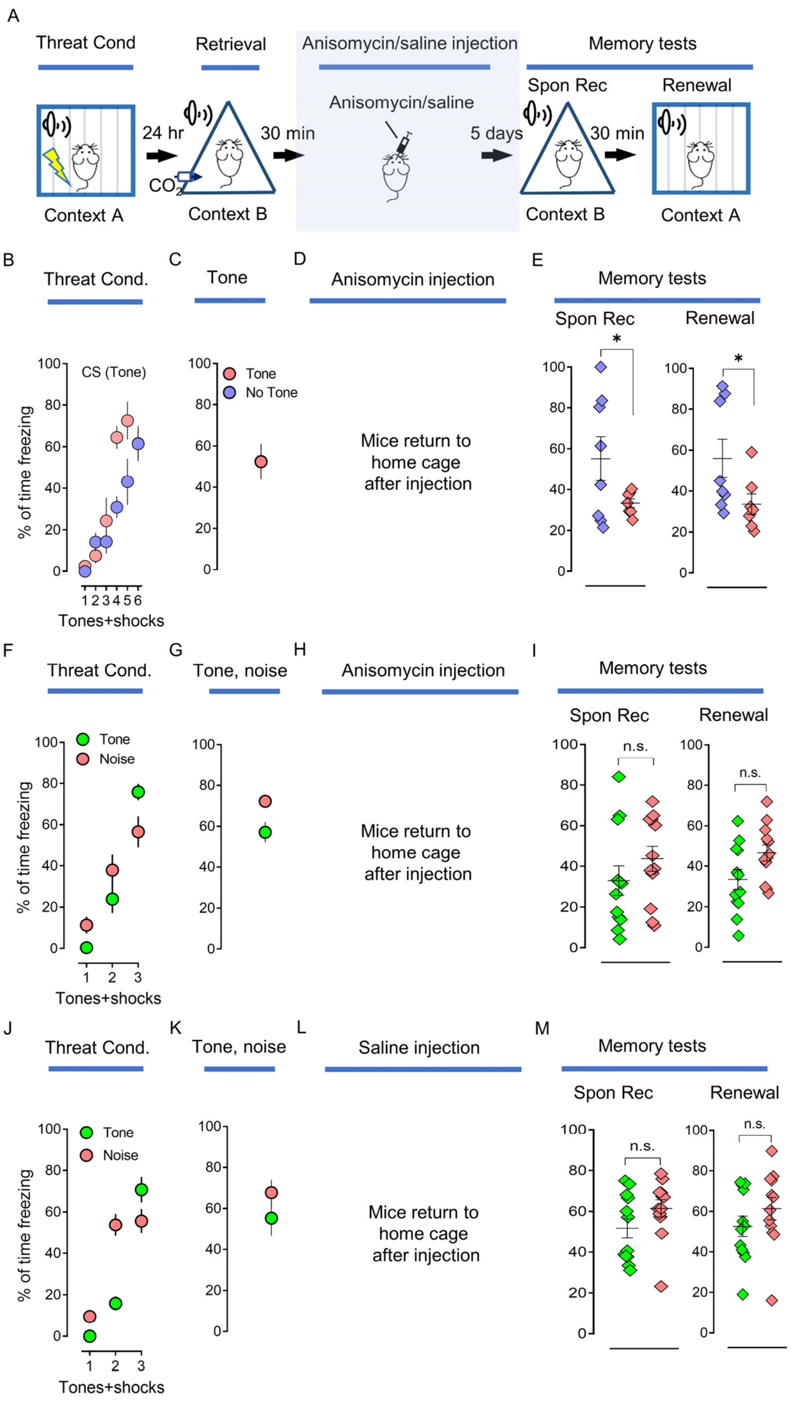

Supplemental Figure 3 Anisomycin disrupts the memory within the reconsolidation window. (A) Schematic protocol for the threat conditioning (pure tone), with or without memory retrieval (pure tone), anisomycin infusion, and memory test-Spon Rec and renewal. **(B-E)** Data are presented by the percentage of freezing time during the tone presentation in threat conditioning **(B)**, with or without retrieval (tone) **(C)**, anisomycin infusion in the amygdala **(D)**, Spon Rec and renewal test with tones **(E)**, n = 8 mice in each group. **(F-I)** Data are presented by the percentage of freezing time during the CSs (tone and noise) in threat conditioning **(F)**, retrievals

(pure tone and white noise) **(G)**, anisomycin injection in the amygdala **(H)**, Spon Rec and renewal with tone and noise **(I)**, n = 12 mice in each group. **(J-M)** Data are presented by the percentage of freezing time during the CSs (tone and noise) in threat conditioning **(J)**, retrievals (pure tone plus CO_2 inhalation and white noise) **(K)**, saline injection in the amygdala **(L)**, Spon Rec and renewal with tone and noise, **(M)**, n = 12 mice in each group. Data are mean ± SEM. 'n.s.' indicates not statistically significant. * indicates $p < 0.05$ by two-tailed paired Student's t-test.

Supplemental Figure 4

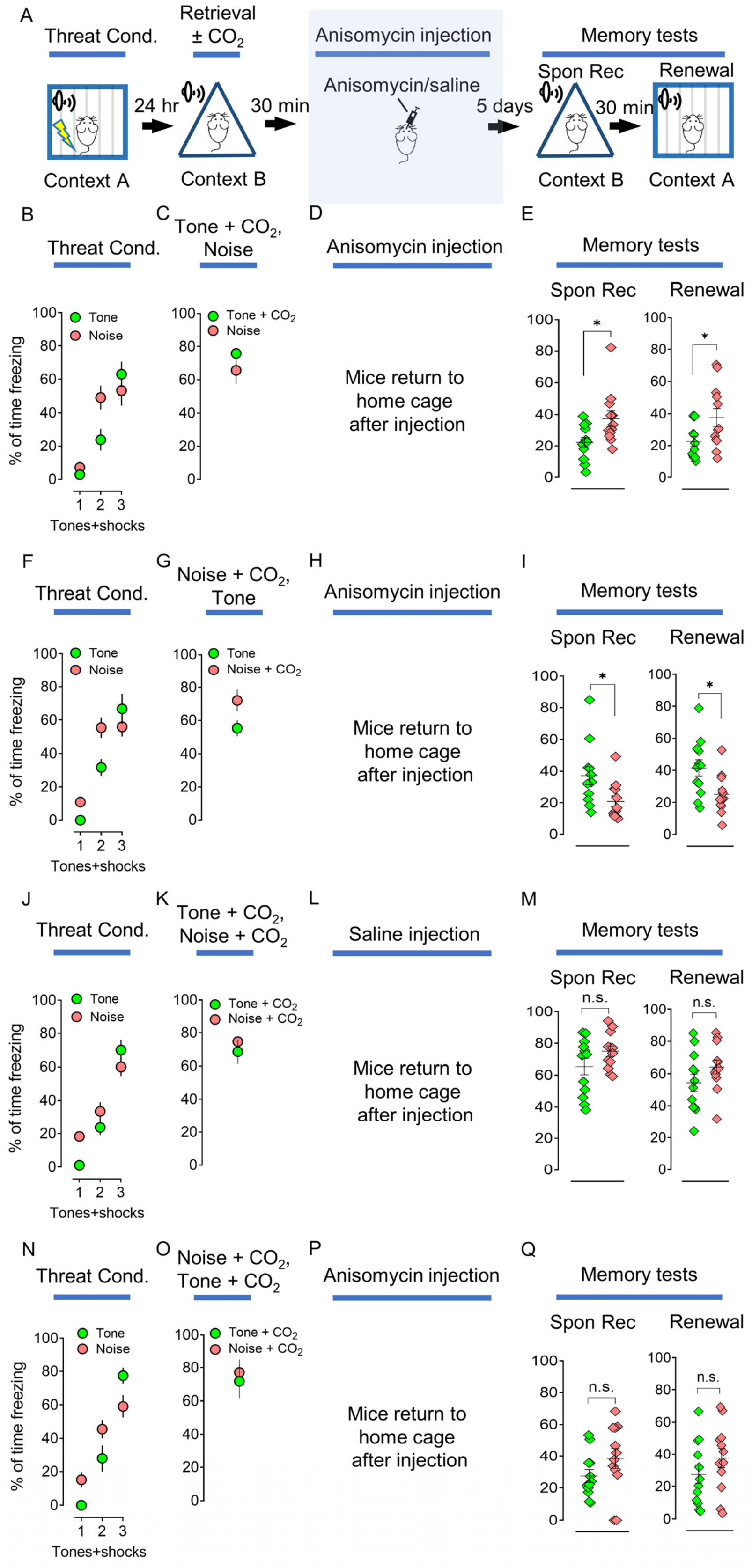

Supplemental Figure 4 CO_2 enhances memory retrieval and potentiates the effects of anisomycin on reconsolidation. (A) Schematic protocol for the threat conditioning (pure tone and white noise), memory retrieval (pure tone and white noise) with or without CO_2 inhalation, anisomycin infusion, and memory test-Spon Rec and renewal. **(B-E)** Data are presented by the percentage of freezing time during the CSs (tone and noise) presentation in threat conditioning **(B)**, retrieval (tone plus CO_2 and noise) **(C)**, anisomycin infusion in the amygdala **(D)**, Spon Rec and renewal test with tone and noise **(E)**. **(F-I)** Data are presented by the percentage of freezing time during the CSs (tone and noise) in threat conditioning **(F)**, retrieval (noise plus CO_2 and tone) **(G)**, anisomycin infusion in the amygdala **(H)**, Spon Rec and renewal with tone and noise **(I)**. **(J-M)** Data are presented by the percentage of freezing time during the CSs (tone and noise) in threat conditioning **(J)**, retrievals (tone and noise) plus CO_2 **(K)**, saline infusion in the amygdala **(L)**, Spon Rec, and renewal with tone and noise **(M)**. **(N-Q)** Data are presented by the percentage of freezing time during the CSs (tone and noise) in threat conditioning **(N)**, retrievals (tone and noise) plus CO_2 **(O)**, anisomycin infusion in the amygdala **(P)**, Spon Rec and renewal with tone and noise **(Q)**. Data are mean ± SEM. n = 12 mice in each group. ‘n.s.’ indicates not statistically significant. * indicate $p < 0.05$ by two-tailed paired Student’s t-test.

Supplemental Figure 5

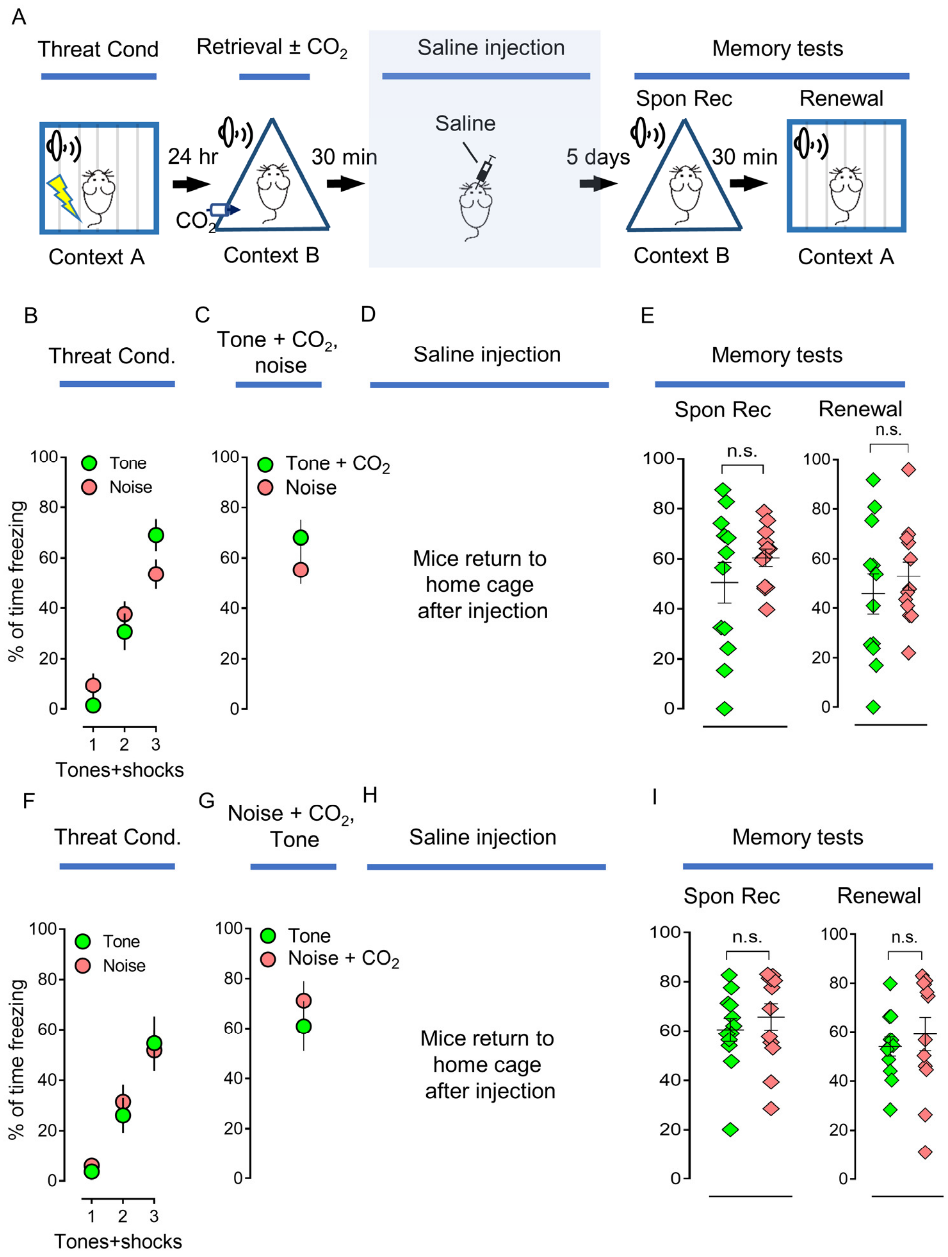

Supplemental Figure 5 CO_2 does not affect memory reconsolidation without anisomycin. (A) Schematic of protocol for the threat conditioning (pure tone and white noise), memory retrieval (pure tone and white noise) with or without CO_2 inhalation, saline infusion, and memory test-Spon Rec and renewal. **(B-E)** Data are presented by the percentage of freezing time during the CSs (tone and noise) presentation in threat conditioning **(B)**, retrieval (tone plus CO_2 and noise) **(C)**, saline infusion in the amygdala **(D)**, Spon Rec, and renewal test with tone and noise **(E)**. **(F-I)** Data are presented by the percentage of freezing time during the CSs (tone and noise) in threat conditioning **(F)**, retrieval (noise plus CO_2 and tone) **(G)**, saline infusion in the amygdala **(H)**, Spon Rec, and renewal with tone and noise **(I)**. Data are mean ± SEM. n = 12 mice in each group. 'n.s.' indicates not statistically significant by two-tailed paired Student's t-test.

Supplemental Figure 6

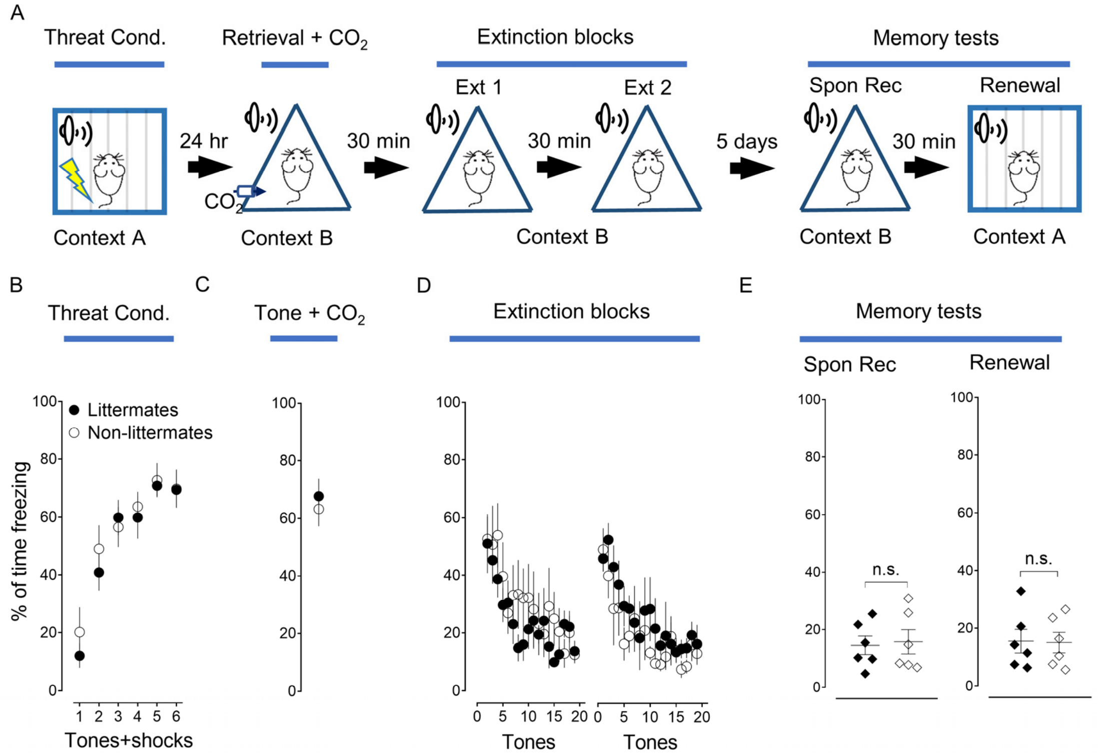

Supplemental Figure 6 Comparison of effects of CO_2 on ASIC1a$^{+/+}$ littermates and non-littermates. (A) Schematic of protocol for the threat conditioning, memory retrieval, extinction, and memory test. **(B-E)** Data are presented by the percentage of freezing time during conditioning, retrieval, extinction, and memory test. Data are mean ± SEM. n = 6 mice in each group. 'n.s.' indicates not statistically significant by two-tailed unpaired Student's t-test.
